# Cardiac Manifestations in Patients with COVID-19: A Scoping Review

**DOI:** 10.5334/gh.1037

**Published:** 2022-01-12

**Authors:** Sasha Peiris, Pedro Ordunez, Donald DiPette, Raj Padwal, Pierre Ambrosi, Joao Toledo, Victoria Stanford, Thiago Lisboa, Sylvain Aldighieri, Ludovic Reveiz

**Affiliations:** 1 Incident Management Systems for COVID-19, Pan American Health Organization, Washington, DC, USA; 2 Health Emergencies Department, Pan American Health Organization, Washington, DC, USA; 3 Noncommunicable Disease and Mental Health Department, Pan American Health Organization, Washington, DC, USA; 4 Department of Medicine, University of South Carolina and University of South Carolina School of Medicine in Columbia, SC, USA; 5 Department of Medicine, University of Alberta, Edmonton, Alberta, CA; 6 Department of Cardiology, Hôpital de la Timone, Marseille –Aix-Marseille Université, Marseille, FR; 7 Evidence and Intelligence for Action in Health Department, Pan American Health Organization, Washington, DC, USA; 8 Instituto de Pesquisa HCOR – Hospital do Coração São Paulo, BR

**Keywords:** COVID-19, Heart, Complications, Heart Diseases, Review

## Abstract

**Background:**

Coronavirus disease 2019 (COVID-19), commonly affects the lungs, but the involvement of other organs, particularly the heart, is highly prevalent as has been reported in several studies. The overall aim of this review was to provide an in-depth description of the available literature related to the cardiac system and COVID-19 infection. It focuses on type and the frequency of cardiac manifestations, clinical parameters and cardiac biomarkers that support the prognosis of COVID-19 patients, and the cardiac adverse events and outcomes related to pharmacotherapy.

**Methods:**

A scoping review was conducted searching Embase, PubMed, Epistomonikos, Medrxiv, BioRxiv databases, up to November 2020, for systematic reviews relevant to cardiac manifestations in adult COVID-19 patients. Relevant articles were screened and extracted to summarize key outcomes and findings.

**Results:**

A total of 63 systematic reviews met the inclusion criteria. The overall frequency of acute cardiac injury ranged from 15% to 33% in the reporting studies. The main cardiac complications were arrhythmias (3.1% to 6.9% in non-severe patients, 33.0% to 48.0% in severe disease), acute coronary syndromes (6% to 33% in severe disease), and myocarditis. Most studies found no association with the use of Renin-angiotensin-aldosterone system inhibitors (RAASI) with COVID-19 outcomes such as susceptibility to infection, hospitalization, severity, and mortality.

**Conclusion:**

This study provided an overview of the several cardiac complications associated with Covid-19. Cardiac injury, arrhythmias, myocarditis, cardiac failure, and acute coronary syndrome, are prevalent and clinically significant and associated with COVID-19 disease severity and mortality. Other studies are needed to clearly identify what is the part of viral heart infection and what is the part of cardiac injury secondary to acute respiratory failure and inflammation. In the therapeutic field, these systematic reviews gave heterogenous results. This underlines the importance of randomized trials to determine the right therapeutic approach.

## Introduction

Coronavirus disease 2019 (COVID-19) pandemic, caused by the Severe Acute Respiratory Syndrome Coronavirus 2 (SARS-CoV-2) has affected over 100 million persons and caused more than 2 million deaths globally [1], one year following its emergence in December 2019, in Wuhan China [2]. COVID-19 presents with a heterogeneous clinical course, ranging from asymptomatic carrier status to a fatal outcome with multi-organ failure [3], or the presence of persistent post COVID-19 conditions [4].

Although the lungs are the most affected organ by COVID-19, the involvement of other organs, particularly the heart, is highly prevalent as has been reported in several studies [5]. Cardiac injury is frequently observed in hospitalized patients with moderate or severe illness secondary to COVID-19, particularly among those with underlying, pre-existing cardiovascular disease (CVD) [6,7,8]. It is important to note that the presence of cardiac injury is linked to a poorer prognosis [5,9,10]. Pre-existing CVD and CVD risk factors have been shown to increase the severity of COVID-19, leading to the aggravation and decompensation of chronic underlying cardiac pathologies, as well as the acute-onset of new cardiac complications [11]. Acute cardiac injury, which may be manifested by myocardial dysfunction, increased blood levels of cardiac troponin, and/or electrocardiographic (ECG) abnormalities, appears to be frequent in hospitalized patients with COVID-19 [12].

Angiotensin converting enzyme-2 (ACE-2) is the host cell receptor responsible for mediating infection by SARS-CoV-2. ACE2 expression is upregulated by Renin-angiotensin-aldosterone system inhibitors (RAASI) that includes ACE inhibitors (ACEis) and angiotensin receptor blockers (ARBs) in the treatment of hypertension. Therefore, this has led to the speculation that patients taking ACEIs or ARBs may be more susceptible to SARS-CoV-2 infection and an increase in severity to more severe manifestations of COVID-19 [13].

## Rationale for the present scoping review

The overall aim of this review is to provide an in-depth description of the available literature related to the cardiac system and COVID-19 infection, as well as to map and synthesize this information. This process could then inform healthcare practitioners, policymakers, and researchers to support evidence-informed decision making.

A preliminary search for existing scoping reviews on the topic was conducted in PubMed, EMBASE, Epistomonikos, and the Cochrane Database of Systematic Reviews in November 2020. Two scoping reviews on this topic were found [14,15]. One focused on the risk of COVID-19 infection in the presence of preexisting CVD and new cardiac manifestations and the other report focused primarily on the pathology and prevalence of cardiac manifestations in COVID-19 infected patients. Both these scoping reviews were conducted in the early stage of the pandemic (April and May 2020) where there were limited reports focusing on the CV manifestations in COVID-19 patients.

The objectives of this scoping review are to update and summarize the existing systematic reviews on the frequency of cardiac manifestations and clinical presentation in COVID-19 patients, the clinical parameters and cardiac biomarkers that support the prognosis of COVID-19 patients, and the cardiac adverse events and outcomes related to pharmacotherapy; to assess the current evidence-based on interventions to prevent or treat cardiac complications in COVID-19 including the use of RAASIs.

## Method

This scoping review follows the framework outlined by Arksey and O’Malley [16] and the adopted, updated recommendation by Levac and colleagues [17], and complies with the Preferred Reporting Items for Systematic Reviews and Meta-analyses extension for scoping reviews (PRISMA-ScR) [18] (S1 File).

To ensure that the scoping review methods were reproducible, transparent, and consistent, a scoping review protocol was developed a *priori* (S2 File). The background and rationale for this scoping review have been described in the protocol. The protocol, list of definitions, search algorithms, location of the repository of relevant articles, and the dataset resulting from this review is available in the supplementary file. The review team consisted of individuals with multi-disciplinary expertise in public health, epidemiology, cardiology, and emergency medicine. This article is based on previously conducted studies and does not contain any studies with human participants or animals performed by any of the authors.

### 1. Identifying the research questions

Consistent with the standard recommendations for scoping reviews, we convened an initial meeting of the core review team members and identified the broad primary research questions: 1) What is the involvement of the cardiac system in adult patients diagnosed with COVID-19 and, 2) What are the interactions between SARS-CoV-2 infection and ACEI and ARB treatment, 3) What are the main pathophysiologic mechanisms of cardiac manifestations in Covid-19.

### 2. Identifying relevant studies

A search strategy was developed by an experienced author (LR) and a librarian (GR) (S1 Table). The search was conducted across Embase, PubMed, Epistomonikos, MedRxiv, BioRxiv databases, from its inception to November 2020; no language restrictions were applied. We manually searched the reference lists of systematic reviews that were included, as well as including relevant studies recommended by experts. Additional searches were performed to ensure that new existing systematic reviews did not importantly modify key findings.

### 3. Study selection

Explicit a *priori* eligibility criteria (S2 File) were applied at title and abstract, and full text screening. The principal inclusion criterion was a systematic review that addressed one or more of the study objectives. Primary peer-reviewed articles and nonpeer -reviewed articles were considered relevant if they addressed one or more aspects of the research question. One reviewer uploaded the literature search results into Microsoft excel®, and screened the titles and abstracts of all references, and full texts of the identified studies. Included studies were examined by two other reviewers to confirm inclusion, extract relevant data, and map them to specific research questions. Exclusion criteria were also recorded.

**Inclusion criteria:** Systematic reviews published from inception to November 2020, on diagnosed COVID-19 adult patients, without restrictions on race, gender, geographical location or setting, reporting: cardiac symptoms/complications; cardiac biomarkers, imaging, clinical management for cardiac complications; cardiac adverse events in those on COVID-19 therapeutics; adverse/improved general and cardiac outcomes in patients on RAASI; and pathophysiology of the cardiovascular system involvement in SAR-CoV-2.

**Exclusion criteria:** Systematic reviews reporting on Kawasaki-like syndromes, multi-system inflammatory syndrome (MISC) related to COVID-19, studies enrolling pediatric samples; studies comparing Covid-19 with previous SARS or Middle East Respiratory Syndrome (MERS) infections; animal studies, in vitro experiments, drug modelling, and other unrelated aspects of COVID-19 research.

### 4. Charting the data

A data extraction form (MS excel) was developed and validated by two reviewers (SP and LR). For each study, we extracted data on source, patient demographic, clinical topic covered and outcomes (S2 File).

### 5. Collating, summarizing, and reporting the results

The key information from each article, as described in the data items section, are reported as a narrative and using descriptive statistics using tables and graphs, with no attempt to aggregate findings from various studies, as this is not the aim of a scoping review. We used the qualitative characteristics and the numerical distribution of mapped evidence addressing each of the research questions to formulate our recommendations for future research.

**Risk of Bias assessment:** Two reviewers (SP and VS) assessed the risk of bias across eligible systematic reviews using the tool ROBIS [19]. Any discrepancy was resolved by a third reviewer (LR).

## Results

**Figure 1:**
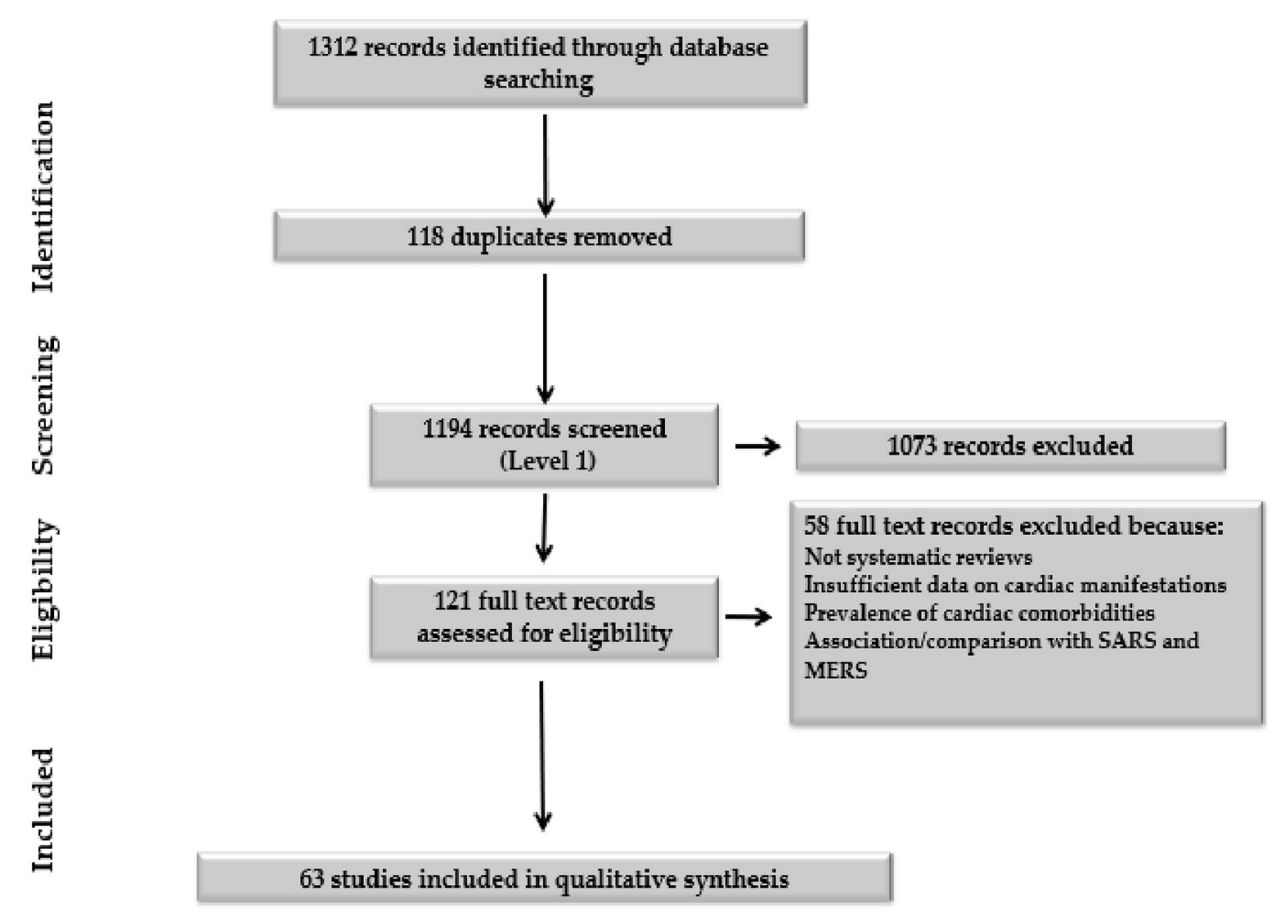
PRISMA flow diagram.

In total we identified 1312 records through database searching and recommendations. Following title and abstract screening (Level 1 screening), 1073 records did not meet the inclusion criteria. The PRISMA flow diagram (***Figure 1***) depicts the detailed process of study selection, and we included a total of 63 systematic reviews.

### Characteristics of included studies

**Figure 2:**
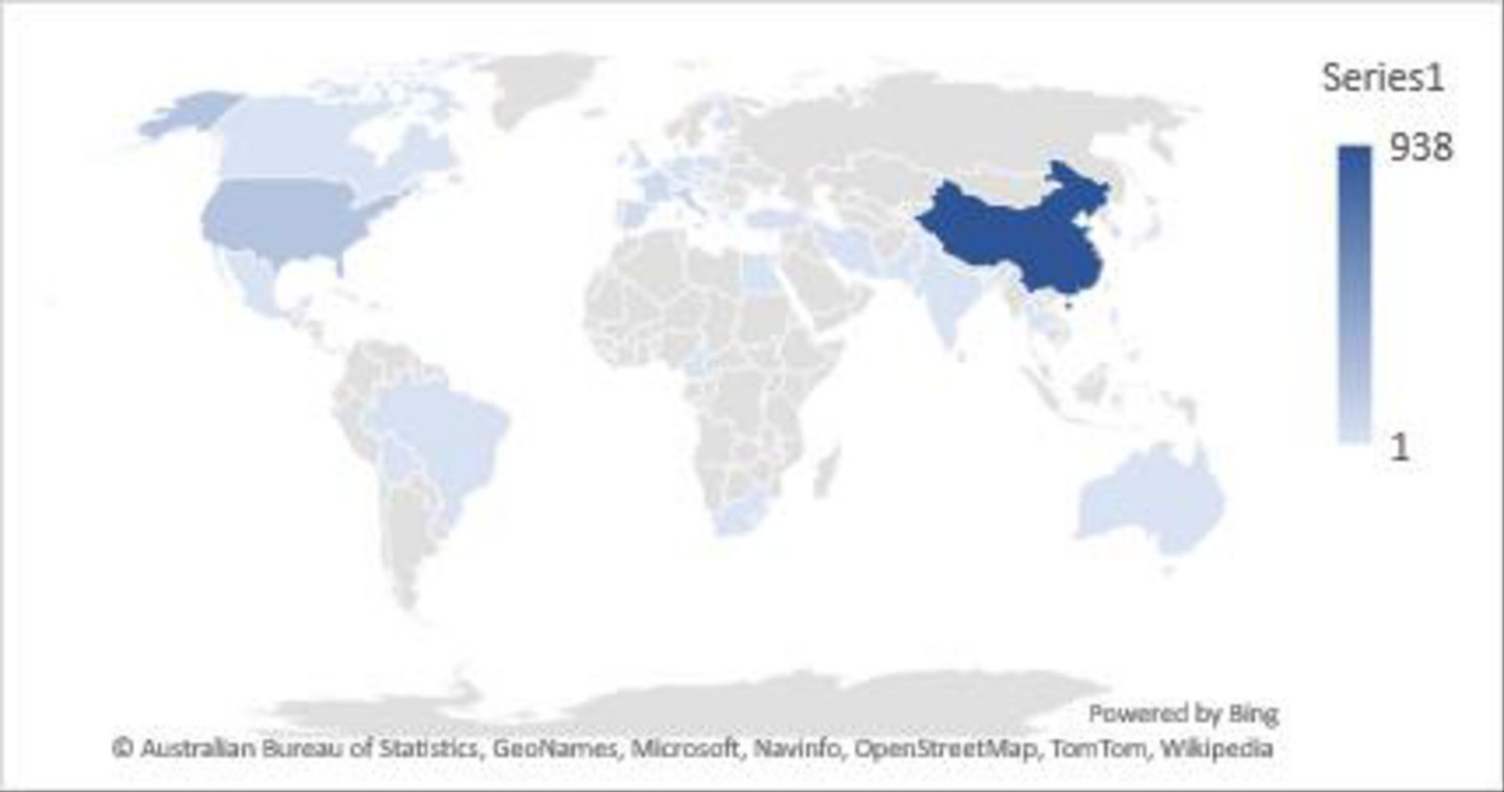
Origin of primary studies included in the reporting systematic reviews.

***Table 1*** summarizes the main characteristics of the 63 systematic reviews (S2 Table includes study title, author, and reference number) that include 46 (73%) peer-reviewed articles, and 17 (27%) non-peer reviewed studies. Fourteen (22.2%) studies were systematic reviews and 49 (77.8%) were systematic reviews with meta-analysis. Fifty-six systematic reviews reported on the origin of the primary studies (n = 1575), most of which come from upper middle-income or high-income countries: China 938 (59.6%), USA 202 (12.8%), or Italy 110 (7.0%) (***Figure 2*** and S3 Table). Eleven studies reported data from a single country, which was from China. Fifty studies reported total patient population, ranging from 12 to 2,065,805 patients. Forty-five (90%) of the 50 reporting studies had more than 1,000 patients each, and 15 (30%) studies reported on more than 10,000 patients per study. The research domains of the included studies were acute cardiac injury, arrhythmias, other CV complications (heart failure, cardiogenic shock, cardiomyopathy, acute coronary syndrome), cardiotoxic therapeutics, RAASI use and pathophysiology of cardiac involvement in COVID-19 patients.

**Table 1:** Characteristics of the included 63 studies and demographics of COVID-19 patients.


STUDY CHARACTERISTICS (N = 63)	

Systematic reviews only	14 (22.2%)

Systematic review and Meta-Analysis	49 (77.8%)

Peer reviewed	46 (73.0%)

Non-peer reviewed	17 (27.0%)

**LOCATION OF PRIMARY STUDIES IN THE REPORTING 56 SYSTEMATIC REVIEWS**	

Single country studies- from China	11/56 (19.6%)

Multi-country studies	45/56 (80.4%)

**DEMOGRAPHICS OF COVID-19 PATIENTS**	

**AGE MEAN/MEDIAN YEARS (RANGE)**	**NUMBER OF STUDIES (N = 63)**

≥50–75	10 (16%)

≥40–87	15 (24%)

≥30–73	5 (8%)

≥20–95	6 (10%)

≥8–109	1 (2%)

Not reported	26 (41%)


The greatest number of cardiac studies are shown to have been published in October (S1 Figure) The highest number of primary cardiac studies of COVID-19 patients included in the systematic reviews throughout the year 2020 is from China (S1 Figure).

From the 33 systematic reviews that reported the gender, 30 (91%) studies had predominant COVID-19 male population (>50%). From the 37 studies reporting the mean or median age of the patients, 25 (67.6%) included patients that were 40 years and above, and 36 (97.2%) included patients that were 20 years and older.

Risk of bias assessment: Of the 63 studies we evaluated, 45 studies had a low risk of bias, while nine studies had a high risk of bias and nine were unclear (S2 Figure).

### 1) What is the involvement of the cardiac system, in adult patients diagnosed with COVID-19

The reported cardiac manifestations in COVID-19 patients are summarized in ***Tables 2*, *3***, and ***4*** (S4 Table and S3 Figure).

**Table 2:** Summary of cardiac complications in COVID-19 patients.


CARDIAC COMPLICATIONS IN COVID-19 PATIENTS	ESTIMATES	REFERENCES

**Acute cardiac injury (ACI) (frequency)**		

Overall frequency **	15% to 33%, and 75% in 1 study	[20,23,25,27,28,45,53,56,57,58,59,60,61]

Patients with CVD and/or in severe disease **	25%, to 33%	[23,25]

In fatal cases	61.6% to 72.6%	[53,59,60]

In patients with Takotsubo syndrome	75%	[20]

Increased risk of ACI in severe disease	(OR) 13.5, 6.6, 6.3	[23,30,31,47,60,62,63]

	(RR) 6.0, 13.8, 8.5, 5.7	

Association of ACI with mortality	(OR) 17.0, 19.6, 20.3, 21.2, 22.5	[23,30,31,33,34,47,54,59,63]

	(RR) 3.8, 4.9, 8.0, 8.5, 8.9	

**ARRHYTHMIA**		

Overall**	0.3% to 44.0%	[21,22,23,24,25,26,27,28,29,41,42,43,61]

Incidence in severe/fatal patients**	33.0% to 48.0%	[21,25,29,30,34]

Incidence in non-severe patients**	3.1% to 6.9%	[21,30,34]

Incidence related to use of HCQ and/or CQ**	0.3% to 44.0%	[41,42,43]

QT prolongation (overall frequency) **	9% to 44%	[29,41,42,43]

Heart failure/shock (frequency)**	3.4% to 23.7%	[23,25,27,28,61]

Cardiac arrest	0.3%, 5.7%	[28,42]

Cardiomyopathy	7%	[25]

ACS/CAD	6.2%, 10%, 33%*	[24,25,28]


OR: odds ratio, RR: Relative risk, HCQ: Hydroxychloroquine, CQ: Chloroquine, ACI: acute cardiac injury, ACS/CAD: Acute coronary syndrome/Coronary artery disease. * Reporting in a case series in a systematic review [24] ** Reporting the lowest and highest proportions.

**Table 3:** Studies addressing acute cardiac injury and myocardial injury in COVID-19 patients.


SOURCE	SAMPLE SIZE	PRE-EXISTING CARDIAC DISEASE IN STUDY POPULATION	ACI/MI FREQUENCY	ACI/MI SEVERE VS NON-SEVERE/MILD DS (OR)/(RR)	ACI/MI AND MORTALITY (OR)/(RR)

Bavishi et al. [45]	11685	NA/NR	20%	–	-

De Lorenzo et al. [56]	1229	NA/NR	16%	–	-

Zou et al. [53]	2224	NA/NR	24%	–	-

Huang et al. [62]	5328	NA/NR	–	OR 13.5 [3.6, 50.5]	-

Li et al. [30]	4189	NA/NR	–	RR 6.0 [3.0, 11.8]	RR 3.8 [2.1, 7.0]

Luo et al. [63]	129380	NA/NR	–	OR 6.6[3.7, 11.6]	OR 17.0 [7.9, 36.4]

Li et al. [64]	3118	45-67%	15%–44%	–	OR 21.2 [10.2, 43.9]

Prastilumkum et al. [57]	8971	NA/NR	20%	–	–

Potere et al. [58]	14866	9.4%	15%	–	–

Zeng et al. [54]	5726	NA/NR	–	–	RR 4.9 [3.8, 6.2]

Zuin et al. [59]	1686	NA/NR	23.90%	–	OR 22.5 [16.1, 31.4]

Santosa et al. [47]	2389	NA/NR	–	RR 13.8 [5.5, 34.5]	RR 8.0 [5.1, 12.3]

Dalia et al. [31]	5967	NA/NR	-	RR 8.5 [3.6, 20.0]	RR 8.5 [3.6, 20.0]

Gu et al. [60]	7679	NA/NR	21%	RR 5.7 [3.7, 8.8]	

Momtazmanesh et al. [23]	11569	NA/NR	25.30%	OR 6.3 [4.2, 9.8]	OR 19.6 [10.3, 37.5]

Shoar et al. [32]	3257	NA/NR	–	–	OR 20.3 [7.8, 53.3]

Martins-Filho et al. [33]	1141	NA/NR	–	–	RR 8.9 [4.2, 19.3]

Amir et al. [65]	29056	NA/NR	33%	–	–

Singh et al. [20]	12	NA/NR	75%	–	–

Sardinha et al. [27]	3316	13.08%	17.09%	–	–

Kunutsor et al. [28]	5815	14.6%	16.30%	–	–

Vakili et al. [61]	6389	NA/NR	15.68%	–	–


NA: Not available, NR: Not reported, ACI: Acute cardiac injury, MI: Myocardial injury, OR: Odds ratio, RR: Relative risk.

**Table 4:** Arrhythmias and QT prolongation in COVID-19 patients in ascending order of publication.


SOURCE	STUDIES/(SAMPLE SIZE)	ARRHYTHMIAS (INCIDENCE)	QT PROLONGATION

Li et al. [30]	22 (4189)	44.4% (severe), 6.9% (non-severe)	–

Jankelson et al. [43]	10 (NR)	7.1% on high dose CQ	10%

Kunutsor et al. [28]	17 (5815)	9.3%	–

Kim et al. [40]	40 (11437)	–	HCQ + AZ OR 1.8 [1.1, 3.3]. There was no significance with HCQ, high-dose HCQ or AZ monotherapy group.

Khadka et al. [39]	6 (NR)	–	HCQ+AZ OR 0.8 [0.6, 1.2]. Increase in critical QTc threshold OR 1.9 [0.8, 4.6] nor absolute ΔQTc ≥60ms OR 2.0 [0.6, 7.0] among HCQ+AZ versus HCQ alone.

Eljaaly et al. [38]	9 (916)	No HCQ associated cardiac toxicity reported	–

Dalia et al. [31]	20 (5967)	Increased risk in non-survivors/severe disease versus survivors/non-severe disease RR 3.6 [2.0, 6.4]	–

Shafi et al. [24]	61 (NR)	14% (AF (7%), VT/VF (5.9%) and AFl)	–

Momtazmanesh et al. [23]	35 (11569)	26.1%	No cardiotoxicity reported

Li et al. [34]	23 (4631)	43.8% (severe), 3.1% (non-severe). Newly occurring arrhythmias were at a higher risk of developing severe disease/ICU admission RR 13.1 [7.0, 24.5]	–

Das et al. [44]	17 (8071)	No significant risk in HCQ group. significantly increased in the HCQ + AZ group	No significant risk of DILQTS in HCQ group vs control. Significantly increased in the HCQ + AZ group

Pranata et al. [21]	4 (784)	19% overall. 48% (severe), 6% (non-severe). increased risk of poor outcome RR 8.0 [3.8, 16.8]	–

Prodromos et al. [36]	25 (NR)	No TDP or related deaths with HCQ + AZT. Found to substantially decrease arrhythmias.	–

Malaty et al. [29]	23 (4911)	6.9% with. HCQ, CQ, AZ. ventricular arrhythmias (VT, VF), atrial arrhythmias (AF, Afl, AT), brady-arrhythmias (AV block, sinus bradycardia).	14.2% overall. 15.9% DILQTS with AZ + HCQ/CQ, 11.44% DILQTS with HCQ or CQ or AZ

Martins-Filho et al. [33]	6 (1141)	Risk for mortality RR 4.9 [1.2, 10.9]	–

Michaud et al. [35]	38 (NR)	–	High to moderate risk of LQTS for CQ, HCQ, Favipiravir, Remdesivir, and LPV/r. Not for AZ.

Shoar et al. [32]	12 (3257)	Risk for mortality OR 22.4 [1.8, 283.6]	–

Vakili et al. [61]	30 (6389)	16.6%	–

Ladapo et al. [37]	5 (5577)	1/936 in HCQ group versus 1/469 control (1/4 reporting study). 0% in 3/4 reporting studies	No HCQ associated LQT reported

Hessami et al. [66]	56 (29056)	Incidence- 11% (overall), 33% (severe Patients). Associated with ICU admission (OR: 22.2, 95%CI 4.5-110.0)	–

Zeng et al. [54]	17 (5726)	CI vs non-CI groups RR 5.7 [0.7, 47.0]	–

Hamam et al. [22]	9 (1445)	19.7%	–

Tleyjeh et al. [42]	19 (5652)	0.3% (overall). 5% incidence of discontinuation of CQ or HCQ due to prolonged QTc or arrhythmias (13 studies of 4334 patients)	9% QTc change form baseline of ≥ 60 ms or QTc ≥ 500 ms, 5% discontinuation of CQ or HCQ due to prolonged QTc or arrhythmias (13 studies of 4334 patients).

Takla et al. [41]	24 (NR)	44% with HCQ and/or CQ, 44% found no evidence of a significant difference, and 11% mixed results	44% greater incidence

Sardinha et al. [27]	12 (3316)	1.77%. (AF most common)	–

Thakkar et al. [26]	101	44%	–


TDP: Torsade de Pointes, OR: odds ratio, RR: relative risk, HCQ: Hydroxychloroquine, CQ: Chloroquine, AZ: Azithromycin, ICU: Intensive care unit, VT: Ventricular tachycardia, VF: Ventricular fibrillation, AF: Atrial fibrillation, Afl: Atrial flutter, AT: Atrial tachycardia, AV block: Atrioventricular block, LPV/r: Lopinavir/Ritonavir, CI: cardiac injury.

The most frequent reported cardiac complications were arrhythmias (S3 Figure and S4 Table), myocarditis (***Table 5***), heart failure, cardiogenic shock, cardiac arrest, and acute coronary syndromes/CAD. One study (n = 12) reporting on 12 patients with Takotsubo syndrome reported acute cardiac injury defined as elevated troponin I, in 75% of the cases [20].

**Table 5:** Myocarditis and COVID-19.


SOURCE	STUDIES/(SAMPLE SIZE)	FREQUENCY/AGE (MEAN RANGE)/PRE-EXISTING DISEASE	CLINICAL SYMPTOMS	ECG	IMAGING – ECHO AND CMRI	INVESTIGATIONS – OTHER	ELEVATED BIOMARKERS	THERAPEUTICS

Sawalha et al. [48]	14 case reports (14)	100%, 21 to 78 years CVD 8%, HTN 33%	Dyspnea 71%, Shock 58%, Chest pain 57%, Cough 67%, fever 75%	diffuse ST-segment elevation 25%, ST-segment depression 25%, T-wave inversion 25%, arrythmias 17%	Reduced LVEF 50%, pericardial effusion 42%, cardiac tamponade 20%, diffuse hypokinesis 30%. Diffuse gadolinium enhancement 100%	CT angiography 17%, invasive coronary angiography 25%, endomyocardial biopsy 7%	Trop. 86%, CKMB 17%, NT-BNP 50%, CRP 100%, IL6 100%	Glucocorticoids, Ig, colchicine. For cytokine storm – Tocilizumab, INF. ECMO (14%)

Kariyana et al. [49]	11 (NR)	12% to 100%, 21 to 74 years	Dyspnea 82%, chest pain/tightness 55%, fever 55%, cough 55%	ST elevation 56%, T wave inversion 33%	Reduced LVEF 67%, pericardial effusion 33%, cardiomegaly 67%. Diffuse gadolinium enhancement 100%	Endomyocardial biopsies	Trop. T 100%, CKMB 100%, NT-BNP 100%	Corticosteroides, LPV/r, HCQ, Ig, tzp, inotropes, vasopressor

Shafi et al. [24]	61 (NR)	12% to 100%, 8 to 79 years	–	–	–	–	–	Steroids, LPV/r, Tocilizumab

Thakkar et al. [26]	101 (NR)	19%–28%, NR	–	–	–	–	–	–


ECHO: Electrocardiogram, LVEF: Left ventricular ejection fraction, CMRI: Cardiac magnetic resonance imaging, CT angiography: computed tomography angiography, CK-MB: Creatine kinase-MB, pro-BNP: pro Brain Natriuretic Peptide, IL-6: inteleuking-6, CRP:C-reactive protein, LPV/r: Lopinavir Ritonavir, HCQ: Hydroxychloroquine, Ig: Immunoglobulin, tzp: piperacillin/tazobactam, ECMO: extracorporeal membrane oxygenation, INF: interferon.

Incidence of arrythmias ranged from 1.8% to 26% in hospitalized COVID-19 patients [21,22,23,24,25,26,27,28,29], while an incidence of 33% to 48% was reported in severe disease [21,25,26,29,30]. Newly occurring arrhythmias in COVID-19 patients predicted severe disease and/or mortality [21,25,30,31,32,33,34]. The most reported arrhythmias were ventricular arrhythmias (ventricular tachycardia, ventricular fibrillation) [24,29], and atrial arrhythmias (atrial fibrillation, atrial flutter) [24,27,29].

Ten studies aimed to report on the cardiac adverse events due to pharmacological treatment for COVID-19 and gave inconsistent results [35,36,37,38,39,40,41,42,43,44]. While three studies reported a significant association between arrythmias and HCQ or AZT [4243^44^], four studies did not [36,37,38,41]. Two studies suggest a probable cardioprotective role of HCQ [36,37] in COVID-19 patients.

Drug induced long QT syndrome (DILQ) was reported with AZT and HCQ/CQ [29,40,44], and monotherapy with COVID-19 repurposed medicines [35,41,42,43,44], while other studies did not [39,40,44].

COVID-19 patients with severe disease and non-survivors had more prominent laboratory abnormalities (S5 Table), specifically cardiac biomarkers including increased levels of Troponin I [23,24,30,31,32,33,45,46,47,48,49,50,51]. Elevated levels of creatine kinase MB isoenzyme (CK-MB) [23,24,30,31,33,34,48,49,50,51,52] NT-BNP [24,30,31,32,33,34,48,49,50,51,53,54,55], lactate dehydrogenase (LDH) [23,32,34,46,51] and D-dimer levels [23,50,51,54] were also reported and associated with disease severity and mortality.

Four studies focused on myocarditis [24,26,48,49]. The general clinical symptoms observed in COVID-19 patients with myocarditis are similar to those observed in a respiratory tract infection such as dyspnea, chest pain, fever, and cough. These patients showed ST elevation (25%–55.6%) and T-wave inversion (25%–33.3%) on ECG, reduced left ventricular ejection fraction (LVEF) (50%–66.7%) on ECHO, and diffuse/late gadolinium enhancement on cardiac MRI (100%). Shafi et al. reported the use of CMRI with gadolinium washout, combined with ECHO findings to confirm myocarditis [24]. Glucocorticoids, immunoglobulins, and antivirals were the most used pharmacotherapy.

### 2) What are the interactions between SARS-CoV-2 infection and ACEI and ARB treatment

Eight (66.7%) out of 12 systematic reviews found no association between the use of ACEI or ARBs and COVID-19 outcomes (***Table 6*** and S6 Table) such as susceptibility to infection, hospitalization, severity and mortality, while some studies found no association even after adjusting for potential confounding factors [67,68,69,70,71,72,73,74]. However, in one study the use of RAASI was found to decrease the length of hospital stay [67]. Other studies including a study in which data was collected on 2,065,805 individuals [75] suggest a protective role of RAASI, decreasing the risk of severe illness and mortality [74,75,76,77,78,79,80].

**Table 6:** Risks associated to the use of RAAS inhibitors in COVID-19 patients.


SOURCE	ACEI/ARB-TESTING COVID-19 POSITIVE	ACEI/ARB-HOSPITALIZATION	ACEI/ARB-SEVERE DISEASE	ACEI/ARB-LENGTH OF HOSPITALIZATION	ACEI/ARB-MORTALITY		

Asiimwe et al. [67]	OR 1.01 [0.93, 1.10]	OR 1.16 [0.80, 1.68]	OR 1.04 [0.76, 1.42]	MD-0.45	OR 0.86 [0.64, 1.15]		

Xu et al. [74]	aOR 1.00 [0.94, 1.05]	–	aOR 0.95 [0.73, 1.24]	–	aOR 0.87 [0.66, 1.14]		

Beressa et al. [68]	–	–	RR 0.92 [0.74, 1.14]	WMD -2.33 [5.60, 0.75]	RR 0.73 [0.63, 0.85]		

De Almeida-Pititto et al. [69]	–	–	OR 0.76 [0.39, 1.49]	–	–		

Baral et al. [70]	–	–	OR 0.833 [0.605, 1.148]	–	OR 0.650 [0.356, 1.187]		

Barochiner et al. [76]	–	–	RR 0.81 [0.63-1.04]	–	RR 0.81 [0.63-1.04]		

Bezabih et al. [77]	–	–	OR 0.84 [0.73, 0.96]	–	OR 0.84 [0.73, 0.96]		

Flacco et al. [71]	–	–	OR 1.00 [0.84, 1.18]	–	OR 0.85 [0.81, 1.03]		

Garg et al. [78]	–	–	OR 1.18 [0.91, 1.54]	–	OR 1.03 [0.69, 1.55]		

Zhang et al. [72]	OR 0.93 [0.85, 1.02]	–	aOR 0.76 [0.52, 1.12]	–	aOR 0.97 [0.77, 1.23]		

Ssentongo et al. [75]	OR 0.93 [0.85, 1.02]	–	–	–	RR 0.65 [0.45, 0.94]		

Kaur et al. [79]	–	OR 2.1 [1.09, 4.05]	OR 1.08 [0.79, 1.46]	–	OR 0.91 [0.65, 1.26]		

Liu X et al. [73]	OR 0.95 [0.89, 1.05]	–	OR 0.75 [0.59, 0.96]	–	OR 0.52 [0.35, 0.79]		

Bin Abdulhak et al. [80]	–	–	–	–	aOR 0.33 [0.22, 0.49]		

**REF. NO.**	**ACEI- TESTING POSITIVE**	**ARB- TESTING POSITIVE**	**ACEI- HOSPITALIZATION, ARB- HOSPITALIZATION**	**ACEI- SEVERE DISEASE**	**ARB- SEVERE DISEASE**	**ACEI- MORTALITY**	**ARB- MORTALITY**

Asiimwe et al. [67]	aOR 0.97 [0.87, 1.09]	aOR 0.90 [0.65, 1.24]	aOR 0.78 [0.47, 1.28], aOR 1.09 [0.67, 1.77]	aOR 0.72 [0.46, 1.13]	aOR 1.12 [0.69, 1.82]	aOR 0.80 [0.46, 1.38]	aOR 1.11 [0.94, 1.32]

Xu J et al. [74]	aOR 0.95 [0.88, 1.02]	aOR 0.97 [0.82, 1.14]	–	aOR 0.81 [0.61, 1.08]	aOR 1.09 [0.76, 1.55]	aOR 0.51 [0.23, 1.12]	aOR 1.63 [0.61, 4.35]

Bezabih et al. [77]	–	–	–	OR 0.77 [0.63, 0.93]	OR 1.13 [0.95, 1.35]	OR 0.77 [0.63, 0.93]	OR 1.13 [0.95, 1.35]

Flacco et al. [71]	–	–	–	OR 0.90 [0.65, 1.26]	OR 0.92 [0.75, 1.12]	OR 0.90 [0.65, 1.26]	OR 0.92 [0.75, 1.12]

Garg et al. [78]	–	–	–	OR 1.34 [0.96, 1.87]	OR 1.25 [0.93, 1.67]	OR 1.07 [0.37, 3.05]	OR 1.07 [0.81, 1.43]

Zhang et al. [72]	aOR 0.90 [0.79, 1.04]	OR 1.12 [0.96, 1.32]	–	OR 0.93 [0.59, 1.48]	OR 0.91 [0.71, 1.17]	–	–

Ssentongo et al. [75]	–	–	–	–	–	RR 0.65 [0.32, 1.30]	–


ACEI: Angiotensin Converting Enzyme Inhibitors, ARB: Angiotensin Receptor Blockers, OR: odds ratio, aOR: adjusted odds ratio, RR: relative risk, WMD: weighted mean difference.

Six of the seven studies reporting subgroup analyses reported no difference in outcomes based on the type of RAASI [67,71,72,74,75,78], while only one study reported that taking ACEIs were better in decreasing the severity and mortality compared with those receiving ARBs [77].

### 3. What are the main pathophysiologic mechanisms of cardiac manifestations in Covid-19?

Five systematic reviews reported on the cardiac pathology in COVID-19 patients (S7 Table) [45,81,82^83^,^84^]. The reported mechanisms of myocardial injury considered plausible were: hyperinflammation and cytokine storm, mediated through pathologic T cells and monocytes, leading to myocarditis; respiratory failure and hypoxemia resulting in damage to cardiac myocytes; downregulation of ACE2 expression and subsequent protective signaling pathways in cardiac myocytes; hypercoagulability and development of coronary microvascular thrombosis; diffuse endothelial injury; and inflammation and/or stress causing coronary plaque rupture or supply-demand mismatch leading to myocardial ischemia/infarction (MI).

## Discussion

In this scoping review, we aimed to collate the results of the systematic reviews on the cardiac involvement in COVID-19, specifically the clinical characteristics, cardiac complications, pharmacotherapy, and pathophysiology.

### Clinical presentation/feature

The most prevalent cardiac presentation in SARS-CoV-2 infection are arrhythmias, heart failure and acute coronary syndromes. These presentations were similar across the global regions; however, the origin of the primary studies was dominated by three countries, China, the USA, and Italy, that were epicenters for Asia, the Americas, and Europe, respectively.

### Cardiac markers

The most widely cited laboratory indicator of acute cardiac injury in COVID-19 is elevated serum levels of troponin I, which is released from damaged cardiomyocytes [85]. Usually, acute cardiac injury was defined as cTn >99th percentile. This definition may be questioned since plasma levels of ultra-sensitive troponins are frequently increased in many settings such as respiratory failure in the absence of acute coronary syndromes or myocarditis [86]. Other cardiac biomarkers, CKMB, BNP and LDH were associated with COVID-19 disease severity and cardiac injury. How these cardiac biomarkers may be used for treatment adaptation needs to be evaluated. An increase in D-dimer levels in these patients may be due to a thrombogenic state caused by the elevated proinflammatory cytokines, suppressing cardiac function directly, damaging endothelial cells and amplifying vascular inflammation [87]. Little is said in these reviews about the role of cardiac infection in the myocardial injury. Recent pathological findings suggest that the SARS-CoV-2 infection of the heart is very uncommon, even in the severe forms [88].

### Myocarditis

It is difficult to estimate true prevalence from the available literature, given that three of the four papers [24,48,49] were case series only (100% prevalence, by definition, because only positive cases are included). The remaining study [26] that included 101 patients reported a prevalence of 19–28%; however, this is likely an overestimate of true prevalence given that it is a selected cohort, and few additional details of the patients were provided. The first cohort of myocarditis patients was reported from China [89], with subsequent case reports and case series from USA and Europe [24,48,49]. Presenting symptoms include fatigue, shortness of breath, and chest pain. Tachyarrhythmias are a potential cause of clinical deterioration, but overall prevalence of this complication remains unclear. Clinical diagnosis is made in by looking for typical myocarditis characteristics (symptoms plus elevated troponin and B-type natriuretic peptide) and myocardial ischemia and cardiomyopathy should also be considered in the differential [90]. Notably, electrocardiographic changes seen in pericarditis (widespread ST segment elevation, t-wave inversion, PR segment depression) may not been seen in myocarditis [91]. Three studies in our review reports the use of echocardiography and cardiac magnetic resonance imaging (CMRI) for the diagnosis of myocarditis [24,48,49], in line with the American Heart Association, which states the requirement of endomyocardial biopsy as a definitive diagnosis and recommends a contrast CT in the absence of a CMRI [92]. No specific, COVID-19-focused myocarditis therapies exist; therefore, patients are largely managed supportively.

### Arrhythmias

Consistently, all the systematic reviews found that cardiac arrhythmias were significantly associated with an increased risk of a poor outcome in COVID-19 patients. COVID-19 patients are prone to the development of arrhythmias, especially supraventricular and ventricular tachycardia, indicative of myocardial injury and or hemodynamic instability and often observed in severe or critical situations. This is line with that was described in patients with severe acute respiratory failure from other origins. This suggests the usefulness of appropriate monitoring by electrocardiogram (ECG) in severe forms.

### COVID-19 therapeutics and arrhythmia/LQTS

Since the outbreak of COVID-19, several medicines have been proposed and are being evaluated in intensive clinical trials in COVID-19 patients. HCQ and CQ are traditional antimalarial and autoimmune disease drugs that have been shown to control the SARS-CoV-2 infection in vitro [93], although the cardiotoxicity of HCQ and CQ should not be neglected. Several systematic reviews included in our study showed an association between these drugs and an increase in serious ventricular arrhythmia [21,22,29]. Since these reviews have been published, several randomized controlled trials have been performed. A recent Cochrane review of these randomized trials [94] concluded that adverse events are tripled in patients on hydroxychloroquine or chloroquine compared to placebo, but very few serious adverse events were found. No significant increase of torsade de pointe incidence was found on hydroxychloroquine.

Furthermore, the combination of HCQ/CQ and azithromycin was shown to be associated with an increased in QT prolongation incidence and fatal cardiac complications in a cardiac-impaired population [39,40,44,95] but not significant with HCQ/CQ monotherapy. Some of the studies reporting LQTS have not deﬁned the methodology used for QTc prolongation. Concomitant medications, electrolyte disturbances, structural heart disease and advancing age may also prolong the QT interval in COVID-19 patients, therefore, regular monitoring the QT interval in hospitalized patients or with the use of biodevices for outpatients maybe helpful in this cohort of patients.

Many of the clinically important therapeutic drug classes for the management of COVID-19 patients such as antihistamines [96], beta-agonist [97], analgesics, antipsychotics [98], are known to cause cardiotoxicity. Given the pivotal role of the immunologic overresponse in COVID-19, anti-inflammatory therapy has been used for treating COVID-19 as well as some of the newer medications such as monoclonal antibodies, tocilizumab, interferon [99]. Unfortunately, our search did not capture systematic reviews reporting on cardiac events related to these medications.

### RAASI

Most of the reviews included in our work did not find any relation between RAASI and COVID-19 outcome. Some suggest a protective effect. Since the publication of these systematic reviews, several RCTs have addressed this question [100,101]. In the largest study, R Lopes et al. [101] found that there was no significant difference in the mean number of days alive and out of the hospital for the patients assigned to discontinue vs continue RAAS inhibitors in a RCT including among 659 patients hospitalized with mild to moderate COVID-19. Given the common use of RAASIs worldwide, based on the available evidence and expert consensus, RAASI should not be systematically interrupted in patients with COVID-19.

### Clinical implications and Research Gaps

Our findings indicate that COVID-19-related cardiac manifestations are common and, in many cases, have clinically important consequences. In terms of current clinical management and future research priorities, our review suggests the following:

1. Clinicians should assess for cardiac disease as clinically indicated. However, whether diagnostic management should change to detect cardiac disease and if this improves outcomes remains unknown.
2. Contemporary guideline recommendations intended for non-COVID-19 patients with cardiac disease should also be applied to the treatment of cardiac disease in patients with COVID-19 unless specific evidence exists to suggest otherwise. This is because current knowledge is at an early stage and there are no targeted therapies available for specific COVID-19 related cardiac complication. Research is needed to verify that this is the best approach and identify focused therapies. In terms of research priorities, it seems prudent to align these with the severity of the potential complication, with acute cardiac injury, arrhythmia, and myocarditis being the most serious. Since recognition of these conditions is comparatively well established (both in patients with COVID-19 and in those without), identification and testing of specific therapies for COVID-19 in general, and cardiac complications, should be prioritized.
  - Given the complex interplay of SARS-CoV-2 with the cardiac system, randomized controlled trials are urgently needed to investigate treatment modalities to reduce the incidence and complications associated with COVID-19 related acute myocardial injury.
  - Despite detection of SARS-CoV-2 RNA/viral particles in myocardial tissue, no clear diagnosis of myocarditis could be made histopathologically [49]. Improved understanding of the pathophysiology of COVID-19-related complications such as myocarditis should be a priority for future research.
  - Potential grants/funding directed to better understand the inter-relationship between cardiac disease and COVID-19, addressing both research platforms and integrated multidisciplinary health systems that deliver care at the same site.
  - Data on the long-term consequences of COVID-19 in the heart have not been studied in the systematic reviews in our scope. There is a need for ongoing investigation of the long-term cardiac consequences of COVID-19, regardless of previous (cardiovascular) diseases or the severity of the disease.
  - There is many anecdotal reports and some studies on the cardiac complications following vaccination for COVID-19. There is a need to synthesize the available data regarding cardiac symptoms in post-vaccinated population.
3. However, until this research is done, patients should not be denied important, evidence-based therapies (e.g., RAASI for hypertension and heart failure) based on speculation.
4. Optimal pathways of care for patients with COVID-19 with cardiac complications need to be identified. Often, important testing is delayed because of isolation requirements and/or concerns regarding transmission risk. Balancing the need to optimize care versus health care provider safety is required.
5. Although is often tempting to initiate unproven therapies in severe cases of COVID-19, clinicians should be alerted to the importance of avoiding non-evidence-based treatments because potential harm may result.

### Limitation

Due to the urgent need for evidence on this topic and limited time, we did not contact authors to clarify the details of primary data when necessary. Studies on this topic are rapidly being conducted and may not have been included in this review when results were published. Many studies have shown the significant association of cardiovascular comorbidity with COVID-19 severity and mortality, therefore was not examined in this review. Given the lack of data in some of the studies, we were unable to gather information on the percentage of COVID-19 inpatients and ambulatory patients. In addition, publication bias may have affected the estimates reported here, as nine studies were assessed as high risk of bias. Limiting the scope to systematic reviews did not allow to consider recent high-quality randomized studies that could address therapeutic questions.

## Conclusion

The systematic reviews included in our work described the cardiac manifestations of COVID 19, mainly acute coronary syndromes, heart failure and arrhythmias. They showed the prognostic importance of these complications. These findings support the implementation of preventive measures particularly for the high-risk groups, early diagnosis, close monitoring, and carefully selected therapeutic to minimize adverse outcomes and cardiac complications including those that arise in the convalescent phase.

More studies are needed to clearly identify what is the part of viral heart infection and what is the part of cardiac injury secondary to acute respiratory failure and inflammation. In the therapeutic field, the included systematic reviews frequently gave heterogenous results. This underlines the importance of randomized trials to determine the right therapeutic approach.

## Additional Files

The additional files for this article can be found as follows:

10.5334/gh.1037.s1 S1 File. Preferred Reporting Items for Systematic reviews and Meta-Analyses extension for Scoping Reviews (PRISMA-ScR) Checklist.

10.5334/gh.1037.s2 S2 File. Protocol.

10.5334/gh.1037.s3 S3 File. Tables s1 to s7 and figures s1 to s3.

## Abbreviations

ACEi, Angiotensin converting enzyme inhibitors; ARB, Angiotensin receptor blockers; CMRI, Cardiac magnetic resonance imaging; CQ, Chloroquine; ECG, Electrocardiogram; HCQ, Hydroxychloroquine; LVEF, Left ventricular ejection fraction; MERS, Middle East Respiratory Syndrome; SARS, Severe Acute Respiratory Syndrome; RAASI, Renin-angiotensin-aldosterone system inhibitors.
