# Supplementary material for: Cardiac Manifestations in Patients with COVID-19: A Scoping Review
Source: Glob Heart. 2022 Jan 12;17(1):2. doi: 10.5334/gh.1037 (PMC8757387; doi:10.5334/gh.1037)
Supplement: S2 File. — Protocol. [file gh-17-1-1037-s2.pdf]

Cardiac manifestations in patients with COVID-19: A scoping review

Protocol

Sasha Peiris[1,2], Pedro Ordunez[3], Donald DiPette[4], Raj Padwal[5], Pierre Ambrosi[6], Joao Toledo[1,2], Victoria Stanford[7], Thiago Lisboa[8], Sylvain Aldighieri[1,2], Ludovic Reveiz[1,7*]

[1] Incident Management Systems for COVID-19, Pan American Health Organization, Washington, DC, USA

[2] Health Emergencies Department, Pan American Health Organization, Washington, DC, USA

[3] Noncommunicable Disease and Mental Health Department, Pan American Health Organization, Washington, DC, USA

[4] Health Sciences Distinguished Professor, University of South Carolina and University of South Carolina School of Medicine in Columbia, SC, USA

[5] Department of Medicine, University of Alberta, Edmonton, Alberta, Canada

[6] Department of Cardiology, Hôpital de la Timone, Marseille –Aix-Marseille Université, Marseille, France

[7] Evidence and Intelligence for Action in Health Department, Pan American Health Organization, Washington, DC, USA

[8] Department of Medicine, Hospital de Clínicas de Porto Alegre, Brazil

*Contact author: reveizl@paho.org

Background:

Coronavirus disease 2019 (COVID-19) due to its fast dissemination and effect on the global health, was declared a pandemic by the World Health Organization (WHO). This disease presents with a heterogeneous clinical course, ranging from asymptomatic carrier status to a fatal outcome with multi-organ failure[i] or the presence of persistent long-term symptoms[ii].

Although the respiratory system is most commonly involved in this disease, involvement of other systems, particularly the heart , has been frequently reported in recent studies [iii], where cardiac injury is observed (>20% cases) in hospitalized patients with moderate or severe COVID-19, particularly among those with underlying cardiovascular (CV) conditions[iv, v]. Pre-existing CV diseases and risk factors are shown to increase the severity of COVID-19, leading to the aggravation and decompensation of chronic underlying cardiac pathologies as well as acute-onset of new cardiac complications[vi]. A large-scale study including 44,672 patients reported that cardiovascular disease was a risk factor for fatality of COVID-19 patients[vii].

Angiotensin-converting enzyme 2 (ACE2), which acts as a receptor for the entry of SARS-CoV-2 is mostly present in the lungs but is also present significantly in the heart[viii], implicating the likely pathophysiology of the associated myocardial injury[ix]. There are multiple pathways to myocardial injury, which includes direct myocyte viral invasion[x], type 1 or 2 myocardial infarction (MI), myocarditis, vasculitis, and other mechanisms related to inflammation, thrombosis, and/or stress[xi]. It should be noted that hypercytokinaemia triggered by the virus, with consequent systemic inflammation may induce myocardial damage[xii]. Depending on the type of myocardial injury, there may be important sequelae if residual inflammation or fibrosis exists.

Acute cardiac injury, manifested by myocardial dysfunction, increased blood levels of cardiac troponin, electrocardiographic abnormalities, appears to be prevalent in subgroups of hospitalized patients with COVID-19[xiii]. However, both the epidemiology across the clinical spectrum of patients with COVID-19 and the mechanisms of acute cardiac injury remain uncertain. Evidence on myocarditis and pericarditis is insufficient or largely based on single-patient case reports[xiv], and heart failure and cardiogenic shock appear to be important causes of morbidity and mortality in COVID-19[xv]. Elevated cardiac Troponin (cTn) levels might be used as a reliable marker of disease severity early in the course of COVID-19[xvi]. Some heterogeneous case reports have shown laboratory and ECG changes that are characteristic of Takotsubo cardiomyopathy[xvii] which is an important differential diagnosis of myocardial injury and it is likely triggered by respiratory infections as well as profound emotional stress caused by the isolation period leading to an excessive release of catecholamines.

Cardiac arrhythmias are a reported CV complication in COVID-19 but with no clear pathophysiological correlation. The potential mechanism seems to be viral myocarditis[xviii] and may also be due to the development of severe hypoxemia and hydro electrolytic disorder in the severe inflammatory phase of the illness[xix]. COVID-19 also predisposes to a prothrombotic state by direct mechanisms (microvasculitis due to viral damage), indirect mechanisms (downregulation of ACE2 receptor, hypoxia, and disseminated intravascular coagulation), and even behavioral mechanisms (bed restriction due to prolonged mechanical ventilation)[xx]

At present, there are limited pharmacotherapies specifically approved for the management of COVID-19 and its associated complications. Clinical consideration has been given to many off label uses of

medications directed to the coronavirus. These agents, such as hydroxychloroquine or chloroquine have the potential for deleterious side effects, including cardiac effects such as QT prolongation, cardiomyopathy, or fluid retention. At present more than 1000 studies addressing various aspects of COVID-19 are registered on ClinicalTrials.gov, including more than 600 interventional studies and randomized clinical trials (RCTs)[xxi]. These promising therapies present important CV side effects. There is also the question on the safety of the use of renin–angiotensin–aldosterone system inhibitors (RAASI)[xxii] and the risk of COVID-19 infection.

As SARS-CoV-2 is a new pathogen, there is very few long-term data on cardiovascular abnormalities or dysrhythmias that may occur in the convalescent phase. Emerging data documenting cardiovascular effect of COVID-19 may highlight the importance of understanding the CV implications, the role of pre-existing CV co-morbidities and the resulting cardiac manifestations such as myocardial injury, myocardial infarctions, arrythmias and heart failure.

Rationale for this study

A preliminary search for existing scoping reviews on the topic has been conducted in PubMed, EMBASE, Epistomonikos and (the Cochrane Database of Systematic Reviews) in October 2020. Two scoping reviews in this topic were found. One assessed the risk of COVID-19 infection in the presence of preexisting CV diseases and new CV manifestations[xxiii] and the other reports, mostly on the pathology and prevalence of cardiac manifestations in COVID-19 patients [xxiv]. Both these scoping reviews were conducted in the early stage of the pandemic (March and May 2020) where there was limited reporting on CV manifestations in COVID-19 patients.

The overall aim of this review is to provide an in-depth description of the available literature related to the cardiac system and COVID-19 infection, as well as to map and synthesize this information. This process could then inform healthcare practitioners, policymakers, and researchers to support evidence-informed decision making, as well as to identify any evidence that is lacking or non-existent to support future research and use of resources for clinical management.

Study objectives: To update and summarize the existing systematic reviews on the frequency of cardiac manifestations and clinical presentation in COVID-19 patients, the clinical presentations, clinical parameters and cardiac biomarkers that support the prognosis of COVID-19 patients, with cardiovascular manifestations and the cardiac adverse events and outcomes related to pharmacotherapy; to assess the current evidence evidence-based on interventions to prevent or treat cardiac complications in COVID-19 including the use of RAASIs; and to identify gaps in the evidence base for tackling pre-existing cardiovascular disease in COVID-19 patients and new cardiac symptoms in COVID-19 patients.

Method

Study design

This scoping review will follow the framework outlined by Arksey and O'Malley[xxv], the adopted updated recommendation by Levac[xxvi] and colleagues and evidence gap mapping as described by the JBI Manual[xxvii]. We will perform a scoping review of the published systematic reviews in the topic of CV and COVID-19. We will also review published literature of RCTs for clinical interventions that cause CV adverse events or adverse outcomes in COVID-19 patients. This review will be conducted to comply with the Preferred Reporting Items for Systematic Reviews and Meta-analyses (PRISMA) extension for scoping reviews (http://prisma-statement.org/Extensions/ScopingReviews).

Search Strategy and study selection

The databases of EMBASE, PubMed, Epistemonikos and LILACs will be searched to identify the relevant systematic reviews from 1 December 2019 to October 2020. No limits will be set on the language or the country where the study was conducted. The input of a research librarian will be sought for refining the search (Annex 1: Search terms). Pre-print databases (Preprints.org, biorxiv.org, medRxiv.org) will also be searched for papers accepted but not yet published and we will scan all retrieved papers for additional references. All retrieved references will be exported to EndNote 20 and deduplicated.

One reviewer (SP) will screen the deduplicated references based on their titles and abstracts. All potentially relevant references will be retrieved in full-text and independently assessed by two reviewers. Any disagreements over eligibility will be resolved by consensus.

Inclusion criteria

Articles fulfilling the following criteria will be considered for inclusion in the review. Full articles only will be included:

Systematic reviews on diagnosed COVID-19 adult patients, without restrictions on race, gender, geographical location or setting, reporting; cardiac symptoms/complications; cardiac biomarkers, imaging, clinical management for cardiac complications; cardiac adverse events in those on COVID-19 therapeutics; adverse/improved general and cardiac outcomes in patients on RAASI; and pathophysiology of the cardiovascular system involvement in SAR-CoV-2.

Exclusion criteria

Systematic reviews reporting on Kawasaki-like syndromes, multi-system inflammatory syndrome (MISC) related to COVID-19, studies enrolling pediatric samples; animal studies, in vitro experiments, drug modelling, and other unrelated aspects of COVID-19 research.

Risk of bias

Two reviewers (SP and VS) will independently assess the risk of bias across eligible systematic reviews using the tool ROBIS[xxviii]. ROBIS is currently aimed at four broad categories of reviews mainly within health care settings: interventions, diagnosis, prognosis, and etiology. Any disagreement between authors over the risk of bias of a publication will be resolved by discussion with a third independent reviewer (LR).

Data extraction/data charting

Title/abstract, full-text screening and extracting data will be conducted and extracted data verified by two reviewers (SP and LR). Charting of data will be shared between two reviewers and cross-checked. Discrepancies will be reviewed in consultation with a third reviewer where required.

A bespoke data extraction form (MS excel) will be developed and validated. All judgements at the full-text screening stage will be collected in this standardized MS Excel ® form. Information extracted from each study includes –

First Author, Year of publication, Received date, Publication date

Title, Type of article, peer reviewed, Country, Language, Duration of literature search, Literature search end date 2020, Search platform/s, Study types, Number of Studies, Sample size

Aims/purpose, Key words, Topic category, Outcome, Male, Age - mean (range), Population

Pre-existing Cardiac disease, Pre-existing heart failure, HTN, Cardiac Signs and symptoms – prevalence, Dyspnea, Chest pain/tightness, Palpitations, Cardiac signs and symptoms - time of admission, Cardiac signs and symptoms - inpatients, Cardiac Screening

Cardiac complication – prevalence, Inclusion criteria, Acute Cardiac injury, ACI Definition, Myocardial injury, Heart Failure, Cardiogenic shock, Cardiac arrest, Myocarditis, Arrythmias, QT prolongation, Tokatsubo syndrome, Infarction, Cardiomyopathy, Acute Coronary Syndrome, Thrombosis,

Diagnosis,

Imaging findings – Xray, Cardiac MRI, Other, ECG findings, Echocardiography, Holter monitoring, Cardiac biomarkers – prevalence, Cardiac biomarkers – Troponin, Cardiac biomarkers - Troponin I, Cardiac biomarkers - Troponin T, Cardiac biomarkers - CK-MB, Cardiac biomarkers - NT-BNP, Cardiac biomarkers – myoglobin, Cardiac biomarkers – LDH, Cardiac biomarkers – BNP, Abnormal Inflammatory markers – prevalence, Inflammatory markers – CRP, Inflammatory markers - IL-6, Inflammatory markers - TNF -a, Inflammatory markers – PCT, ESR, CK, D-dimer, ferritin

Management, COVID therapeutics, CV therapeutics, ACEI/ARB, Prophylactic anticoagulant, Therapeutic anticoagulant, Vasopressors, Inotropes/ diuresis , Anti-inflammatory, GDMT, MCS, ECMO

Drug adverse effects, Duration of the intervention, severe disease/ICU, Discharge/inpatient/death,

Length of hospital stay, Discharge rate, Mortality, Follow up, Pathophysiology

Strategy for data synthesis

The data extracted from eligible papers will be analyzed in MS Excel ®. Descriptive statistics such as mean, median, range, relative frequencies, standard deviations, percentile ranks will be used in this review whenever applicable. We will be exploring percentage of COVID-19 patients who presented with and/or developed cardiovascular complications during hospital stay, most frequently observed CV symptom, most frequently used medicines for COVID-19 patients, range of prevalence for CV symptoms and signs, laboratory and imaging findings, and clinical outcomes described, percentage of COVID-19 patients having underlying CV diseases, average length of stay in hospital, average discharge rate and average mortality rate. Likewise, we will be conducting descriptive analysis (average, frequency and percentage) for the data extracted from the papers related to COVID-19 and therapeutics.

Presentation of results and evidence mapping

Tabular presentation, graphical forms and figures where best suitable will be accompanied by a narrative synthesis in the review text. (e.g. "intervention type", "population", "sample size", "duration of intervention", "aims", "methodology adopted", "key findings", and "gaps in the research mapped)

Dissemination of results:

The completed review will be published in a peer-reviewed journal.

Contact details for further information:

Dr Ludovic Reveiz (reveizl@paho.org)

COVID-19 Incident Management System Team, Pan American Health Organization.

References:

---

[i] Lai CC, Liu YH, Wang CY, et al. Asymptomatic carrier state, acute respiratory disease, and pneumonia due to severe acute respiratory syndrome coronavirus 2 (SARS-CoV-2): Facts and myths. J Microbiol Immunol Infect. 2020.

[ii] Rubin R. As Their Numbers Grow, COVID-19 "Long Haulers" Stump Experts. JAMA. 2020;324(14):1381–1383. doi:10.1001/jama.2020.17709

[iii] Zheng Y-Y, Ma Y-T, Zhang J-Y, Xie X (2020) COVID-19 and the cardiovascular system. Nature Reviews Cardiology. 17:259–260

[iv] Huang C, Wang Y, Li X, et al. Clinical features of patients infected with 2019 novel coronavirus in Wuhan, China. Lancet. 2020;395(10223):497-506. doi:10.1016/S0140-6736(20)30183-5

[v] Driggin E, Madhavan MV, Bikdeli B, et al. Cardiovascular considerations for patients, health care workers, and health systems during the COVID-19 pandemic. J Am Coll Cardiol. 2020;75(18):2352-2371. doi:10.1016/j.jacc.2020.03.031

[vi] Wu Z, McGoogan JM. Characteristics of and important lessons from the coronavirus disease 2019 (COVID-19) outbreak in China. JAMA. 2020;323:1239–42.

[vii] Deng G, Yin M, Chen X, Zeng F. Clinical determinants for fatality of 44,672
patients with COVID-19. Critical care (London, England). 2020;24(1):179.

[viii] Hoffmann M, Kleine-Weber H, Schroeder S, Krüger N, Herrler T, Erichsen S, et al. SARS-CoV-2 cell entry depends on ACE2 and TMPRSS2 and is blocked by a clinically proven protease inhibitor. Cell. 2020;181:271–80.

[ix] Chen L., Li X., Chen M., Feng Y., Xiong C. The ACE2 expression in human heart indicates new potential mechanism of heart injury among patients infected with SARS-CoV-2. Cardiovasc Res. 2020;116:1097–1100.

[x] Lindner D, Fitzek A, Bräuninger H, et al. Association of cardiac infection with SARS-CoV-2 in confirmed COVID-19 autopsy cases. JAMA Cardiol. 2020. doi:10.1001/jamacardio.2020.3551

[xi] Mitrani RD, Dabas N, Goldberger JJ. COVID-19 cardiac injury: Implications for long-term surveillance and outcomes in survivors [published online ahead of print, 2020 Jun 26]. *Heart Rhythm*. 2020;S1547-5271(20)30625-1. doi:10.1016/j.hrthm.2020.06.026

[xii] Ye Q, Wang B, Mao J. The pathogenesis and treatment of the 'Cytokine Storm' in COVID-19. J Infect 2020;80:607–13.

[xiii] S. Shi, M. Qin, B. Shen, et al. Association of cardiac injury with mortality in hospitalized patients with COVID-19 in Wuhan China, JAMA Cardiology (2020)
[xiv] H. Hu, F. Ma, X. Wei, et al. Coronavirus fulminant myocarditis saved with glucocorticoid and human immunoglobulin Eur Heart J (2020), Article ehaa190, 10.1093/eurheartj/ehaa190
[xv] Chatrath, N., Kaza, N., Pabari, P. A., Fox, K., Mayet, J., Barton, C., Cole, G. D., and Plymen, C. M. (2020) The effect of concomitant COVID-19 infection on outcomes in patients hospitalized with heart failure. ESC Heart Failure, https://doi.org/10.1002/ehf2.13059.
[xvi] Aikawa T, Takagi H, Ishikawa K, Kuno T. Myocardial injury characterized by elevated cardiac troponin and in-hospital mortality of COVID-19: An insight from a meta-analysis [published online ahead of print, 2020 Jun 2]. J Med Virol. 2020;10.1002/jmv.26108. doi:10.1002/jmv.26108
[xvii] Siripanthong B, Nazarian S, Muser D, Deo R, Santangeli P, Mohammed Y et al. Recognizing COVID-19-related myocarditis: the possible pathophysiology and proposed guideline for diagnosis and management. Heart Rhythm. 2020;S1547-5271:30422–7.
[xviii] Siripanthong B, Nazarian S, Muser D, Deo R, Santangeli P, Mohammed Y et al. Recognizing COVID-19-related myocarditis: the possible pathophysiology and proposed guideline for diagnosis and management. Heart Rhythm. 2020;S1547-5271:30422–7.
[xix] Lakkireddy DR, Chung MK, Gopinathannair R, Patton KK, Gluckman TJ, Turagam M, et al. Guidance for cardiac electrophysiology during the coronavirus (COVID-19) pandemic from the Heart Rhythm Society COVID-19 Task Force; Electrophysiology Section of the American College of Cardiology; and the Electrocardiography and Arrhythmias Committee of the Council on Clinical Cardiology, American Heart Association. Heart Rhythm 2020; S1547-5271:30289–7.
[xx] Bikdeli B, Madhavan MV, Jimenez D, Chuich T, Dreyfus I, Driggin E, et al. COVID-19 and thrombotic or thromboembolic disease: implications for prevention, antithrombotic therapy, and follow-up. J Am Coll Cardiol 2020;75:2950–73.
[xxi] US National Library of Medicine. ClinicalTrials.gov. Accessed April 30, 2020. https://clinicaltrials.gov/
Comment
[xxii] Clerkin KJ, Fried JA, Raikhelkar J, Sayer G, Griffin JM, Masoumi A, et al. Coronavirus disease 2019 (COVID-19) and cardiovascular disease. Circulation 2020;141:1648–55.
[xxiiixxiii] Yahia F, Zakhama L, Ben Abdelaziz A. COVID-19 and Cardiovascular diseases. Scoping review study. Tunis Med. 2020;98(4):283-294.
[xxiv] Ho JS, Tambyah PA, Ho AF, Chan MY, Sia CH. Effect of coronavirus infection on the human heart: A scoping review. Eur J Prev Cardiol. 2020;27(11):1136-1148. doi:10.1177/2047487320925965
[xxv] Arksey HOML. Scoping studies: towards a methodological framework. Int J Soc Res Method. 2005;8:19–32. doi: 10.1080/1364557032000119616.
[xxvi] Levac D, Colquhoun H, O'Brien KK. Scoping studies: advancing the methodology. Implement Sci. 2010;5:69. doi: 10.1186/1748-5908-5-69.
[xxvii] Peters MDJ, Godfrey C, McInerney P, Munn Z, Tricco AC, Khalil, H. Chapter 11: Scoping Reviews (2020 version). In: Aromataris E, Munn Z (Editors). JBI Manual for Evidence Synthesis, JBI, 2020. Available from https://synthesismanual.jbi.global. https://doi.org/10.46658/JBIMES-20-12
[xxviii] Whiting P, Savović J, Higgins JP, et al. ROBIS: A new tool to assess risk of bias in systematic reviews was developed. *J Clin Epidemiol*. 2016;69:225-234. doi:10.1016/j.jclinepi.2015.06.005
