# Supplementary material for: Cardiac Manifestations in Patients with COVID-19: A Scoping Review
Source: Glob Heart. 2022 Jan 12;17(1):2. doi: 10.5334/gh.1037 (PMC8757387; doi:10.5334/gh.1037)
Supplement: S3 File. — Tables s1 to s7 and figures s1 to s3. [file gh-17-1-1037-s3.pdf]

Cardiac manifestations in patients with COVID-19: A scoping review

Supplementary File 3

S1 Table. Literature search performed using EMBASE, Epistemonikos, PubMed, MedRxiv, BioRxiv

Titles/abstracts were identified from publications using Embase (Embase + Medline), PubMed, MedRxiv and Epistemonikos, full text reviewed, and 63 publications were deemed relevant. Also included were manual searches recommended by experts and references from the included systematic reviews related.

1. EMBASE 11/03/2020

Search	Terms	Results
#1	(heart:ti,ab,kw OR 'heart disease':ti,ab,kw OR cardiovascular:ti,ab,kw OR 'cardiovascular system':ti,ab,kw OR cardiac:ti,ab,kw OR arrythmias:ti,ab,kw OR arrythmia:ti,ab,kw OR dysrhythmia:ti,ab,kw OR atrial:ti,ab,kw OR fibrillation:ti,ab,kw OR ventricular:ti,ab,kw OR myocardial:ti,ab,kw OR coronary:ti,ab,kw OR myocarditis:ti,ab,kw OR cardiogenic:ti,ab,kw OR cardiomyopathy:ti,ab,kw OR 'qt prolongation':ti,ab,kw OR flutter:ti,ab,kw OR parasystole:ti,ab,kw OR cardiomegaly:ti,ab,kw OR endocarditis:ti,ab,kw OR 'pericardial disease':ti,ab,kw OR pericarditis:ti,ab,kw OR pneumopericardium:ti,ab,kw OR aortic:ti,ab,kw OR endocardium:ti,ab,kw OR endomyocardial:ti,ab,kw OR infarction:ti,ab,kw) AND [systematic review]/lim AND [2019-2020]/py	9,772
#2	('coronavirus disease 2019':ti,ab,kw OR covid:ti,ab,kw OR 'severe acute respiratory syndrome coronavirus 2':ti,ab,kw) AND [systematic review]/lim AND [1-12-2019]/sd NOT [4-11-2020]/sd	1657
#3	#1 AND #2	172

2. Pubmed 11/03/2020

Search	Terms	Results
#1	(((((((((((((((((((((((((((((Heart[MeSH Terms]) OR (Cardiac[MeSH Terms])) OR (cardiovascular system[MeSH Terms])) OR (heart[Title/Abstract])) OR (heart disease[Title/Abstract])) OR (cardiac[Title/Abstract])) OR (cardiovascular[Title/Abstract])) OR (arrythmia[Title/Abstract])) OR (arrythmias[Title/Abstract])) OR (dysrhytmias[Title/Abstract])) OR (atrial[Title/Abstract])) OR (fibrillation[Title/Abstract])) OR (ventricular[Title/Abstract])) OR (myocardial[Title/Abstract])) OR (coronary[Title/Abstract])) OR (myocarditis[Title/Abstract])) OR (cardiogenic[Title/Abstract])) OR (cardiomyopathy[Title/Abstract])) OR (Long QT[Title/Abstract])) OR (QT prolongation[Title/Abstract])) OR (flutter[Title/Abstract])) OR (parasystole[Title/Abstract])) OR (cardiomegaly[Title/Abstract])) OR (endocarditis[Title/Abstract])) OR (pericardial[Title/Abstract])) OR (pericardial disease[Title/Abstract])) OR (pericarditis[Title/Abstract])) OR (pneumopericardium[Title/Abstract])) OR (aortic[Title/Abstract])) OR (endocardium[Title/Abstract])) OR (endomyocardial[Title/Abstract])) OR (infarction[Title/Abstract])	2,788,148
#2	((coronavirus disease 2019[Title/Abstract]) OR (covid[Title/Abstract])) OR (severe acute respiratory syndrome coronavirus 2[Title/Abstract])	63,507
#3	#1 AND #2	4562
#4	Filters - Systematic reviews	118

3. Epistemonikos – 11/15/2020 – 57 SR results

4. MedRxiv, BioRxiv – 11/15/2020 - 927 results
5. Embase - 11/22/2020 – 5 results (duplicates)
6. Epistomonikos 11/22/2020 – 10
7. EMBASE – 11/22/2020 – 23 SR results

Search	Terms	Results
#1	('coronavirus disease 2019':ti,ab,kw OR covid:ti,ab,kw OR 'severe acute respiratory syndrome coronavirus 2':ti,ab,kw) AND [1-12-2019]/sd NOT [23-11-2020]/sd	65516
#2	(heart:ti,ab,kw OR 'heart disease':ti,ab,kw OR cardiovascular:ti,ab,kw OR 'cardiovascular system':ti,ab,kw OR cardiac:ti,ab,kw OR arrythmia:ti,ab,kw OR dysrhythmia:ti,ab,kw OR 'atrial fibrillation':ti,ab,kw OR ventricular:ti,ab,kw OR myocardial:ti,ab,kw OR coronary:ti,ab,kw OR myocarditis:ti,ab,kw OR cardiogenic:ti,ab,kw OR cardiomyopathy:ti,ab,kw OR 'qt prolongation':ti,ab,kw OR flutter:ti,ab,kw OR parasystole:ti,ab,kw OR cardiomegaly:ti,ab,kw OR endocarditis:ti,ab,kw OR 'pericardial disease':ti,ab,kw OR pericarditis:ti,ab,kw OR pneumopericardium:ti,ab,kw OR aortic:ti,ab,kw OR endocardium:ti,ab,kw OR 'endocardial disease':ti,ab,kw OR infarction:ti,ab,kw) AND [systematic review]/lim AND [1-12-2019]/sd NOT [23-11-2020]/sd	6806
#3	(pathology:ti,ab,kw OR pathophysiology:ti,ab,kw OR pathogenesis:ti,ab,kw OR 'pathological anatomy':ti,ab,kw) AND [systematic review]/lim AND [1-12-2019]/sd NOT [23-11-2020]/sd	2278
#4	#1 AND #2 AND #3	23

S2 Table. Study reference number, title and author of the 63 included systematic reviews

Ref. No.	First Author	Title
1	Bavishi et al.	Acute myocardial injury in patients hospitalized with COVID-19 infection: A review
2	De Lorenzo et al.	Acute cardiac injury in patients with COVID-19
3	Fengwei et al.	Cardiac Injury and COVID-19: A Systematic Review and Meta-analysis
4	Huang er al.	Clinical features of severe patients infected with 2019 novel coronavirus: A systematic review and meta-analysis
5	Li J-W et al.	The impact of 2019 novel coronavirus on heart injury: A Systematic review and Meta-analysis
6	Li et al.	Cardiac injury associated with severe disease or ICU admission and death in hospitalized patients with COVID-19: A meta-analysis and systematic review
7	Luo et al.	The potential association between common comorbidities and severity and mortality of coronavirus disease 2019: A pooled analysis
8	Li et al.	Impact of cardiovascular disease and cardiac injury on in-hospital mortality in patients with COVID-19: A systematic review and meta-analysis
9	Prasitlumkum et al.	Incidence of Myocardial Injury in COVID-19-Infected Patients: A Systematic Review and Meta-Analysis
10	Parohan et al.	Cardiac injury is associated with severe outcome and death in patients with Coronavirus disease 2019 (COVID-19) infection: A systematic review and meta-analysis of observational studies
11	Potere et al.	Acute complications and mortality in hospitalized patients with coronavirus disease 2019: A systematic review and meta-analysis
12	Zeng et al.	Clinical characteristics of covid-19 with cardiac injury: a systematic review and meta-analysis
13	Zuin et al.	Incidence and mortality risk in coronavirus disease 2019 patients complicated by acute cardiac injury: systematic review and meta-analysis
14	Santosa et al.	Cardiac injury is associated with mortality and critically ill pneumonia in COVID-19: A meta-analysis
15	Dalia et al.	Impact of Congestive Heart Failure and Role of Cardiac Biomarkers in COVID-19 patients: A Systematic Review and Meta-Analysis
16	Gu et al.	Incidence of myocardial injury in coronavirus disease 2019 (COVID-19): a pooled analysis of 7,679 patients from 53 studies
17	Pranata et al.	Incidence and impact of cardiac arrhythmias in coronavirus disease 2019 (COVID-19): A systematic review and meta-analysis
18	Hamam et al.	Cardiac Arrhythmias in Patients with COVID-19: A Systematic review and Meta-analysis
19	Malaty et al.	Incidence and treatment of arrhythmias secondary to coronavirus infection in humans: a systematic review
20	Sawalha et al.	Systematic review of COVID-19 related myocarditis: Insights on management and outcome
21	Kariyana et al.	A Systematic Review of COVID-19 and Myocarditis
22	Momtazmanesh et al.	Cardiovascular disease in COVID-19: a systematic review and meta-analysis of 10,898 patients and proposal of a triage risk stratification tool

23	Pranata et al.	Elevated N-Terminal pro-brain natriuretic peptide is associated with increased mortality in patients with COVID-19: Systematic review and meta-Analysis
24	Shafi et al.	Cardiac manifestations in COVID-19 patients—A systematic review
25	Shoar et al.	Meta-analysis of Cardiovascular Events and Related Biomarkers Comparing Survivors Versus Non-survivors in Patients With COVID-19
26	Tian et al.	Predictors of mortality in hospitalized COVID-19 patients: A systematic review and meta-analysis
27	Walker et al.	Assessing the Elevation of Cardiac Biomarkers and the Severity of COVID-19 Infection: A Meta-analysis
28	Martins-Filho et al.	Myocardial injury biomarkers and cardiac complications associated with mortality in patients with covid-19
29	Pillai et al.	COVID-19 and Major Organ Thromboembolism: Manifestations in Neurovascular and Cardiovascular Systems
30	Trypsteen et al.	On the whereabouts of SARS-CoV-2 in the human body: A systematic review
31	Kordzadeh-Kermani et al.	Pathogenesis, clinical manifestations and complications of coronavirus disease 2019 (COVID-19)
32	Deshmukh et al.	Histopathological observations in COVID-19: a systematic review
33	Hessami et al.	Cardiovascular Diseases and COVID-19 Mortality and Intensive Care Unit Admission: A Systematic Review and Meta-analysis
34	Singh et al.	Takotsubo Syndrome in Patients with COVID-19: a Systematic Review of Published Cases
35	Thakkar et al.	A Systematic Review of the Cardiovascular Manifestations and Outcomes in the Setting of Coronavirus-19 Disease
36	Sardinha et al.	The occurrence of cardiovascular complications associated with SARS-CoV-2 infection: a systematic review
37	Kunutsor et al.	Cardiovascular complications in COVID-19: A systematic review and meta-analysis
38	Das et al.	Efficacy and Safety of Anti-malarial Drugs (Chloroquine and Hydroxy-Chloroquine) in Treatment of COVID-19 Infection: A Systematic Review and Meta-Analysis
39	Michaud et al.	Risk assessment of drug-induced Long QT Syndrome for some COVID-19 repurposed drugs
40	Prodromos et al.	Hydroxychloroquine is protective to the heart, not harmful: a systematic review
41	Ladapo et al.	Randomized Controlled Trials of Early Ambulatory Hydroxychloroquine in the Prevention of COVID-19 Infection, Hospitalization, and Death: Meta-Analysis
42	Eljaay et al.	Hydroxychloroquine safety: A meta-analysis of randomized controlled trials
43	Khadka et al.	Is hydroxychloroquine with macrolide a good combination in COVID-19 compared to hydroxychloroquine alone from cardiac perspective? A systematic review and meta-analysis
44	Kim et al.	Comparative Efficacy and Safety of Pharmacological Interventions for the Treatment of COVID-19: A Systematic Review and Network Meta-Analysis of Confounder-Adjusted 36813 Hospitalized Patients
45	Takla et al.	Chloroquine, hydroxychloroquine, and COVID-19: systematic review and narrative synthesis of efficacy and safety
46	Tleyjeh et al.	The Cardiac Toxicity of Chloroquine or Hydroxychloroquine in COVID-19 Patients: A Systematic Review and Meta-regression Analysis
47	Jankelson et al.	QT prolongation, torsades de pointes, and sudden death with short courses of chloroquine or hydroxychloroquine as used in COVID-19: A systematic review

48	Asiimwe et al.	Cardiovascular drugs and COVID-19 clinical outcomes: a living systematic review and meta-analysis
49	Xu et al.	The Effect of Prior ACEI/ARB Treatment on COVID-19 Susceptibility and Outcome: A Systematic Review and Meta-Analysis
50	Beressa et al.	Effect of Renin-Angiotensin-Aldosterone System inhibitors on outcomes of COVID-19 patients with hypertension: Systematic review and Meta-analysis
51	Almeida-Pititto et al.	Severity and mortality of COVID 19 in patients with diabetes, hypertension and cardiovascular disease: A meta-analysis
52	Baral et al.	Impact of hospitalised patients with COVID-19 taking Renin-Angiotensin-Aldosterone System inhibitors: a systematic review and meta-analysis
53	Barochiner et al.	Use of inhibitors of the renin angiotensin system and COVID-19 prognosis: a systematic review and meta-analysis
54	Bezabih et al.	Comparison of renin-angiotensin-aldosterone system inhibitors with other antihypertensives in association with coronavirus disease-19 clinical outcomes: systematic review and meta-analysis
55	Flacco et al.	Treatment with ACE inhibitors or ARBs and risk of severe/lethal COVID-19: A meta-analysis
56	Garg et al.	Association of Renin Angiotensin System Blockers with Outcomes in Patients with Covid-19: A Systematic Review and Meta-analysis
57	Zhang et al.	Renin Angiotensin System Inhibition and Susceptibility and Outcomes from COVID-19: A Systematic Review and Meta-analysis of 69,200 COVID-19 Patients
58	Ssentongo et al.	Renin-angiotensin-aldosterone system inhibitors and mortality in patients with hypertension hospitalized for COVID-19: a systematic review and meta-analysis
59	Kaur et al.	RAAS blockers and region-specific variations in COVID-19 outcomes: findings from a systematic review and meta-analysis
60	Liu et al.	Association of Renin-Angiotensin-Aldosterone System Inhibition with Risk of COVID-19, Inflammation Level Severity and Death in Patients With COVID-19: A Rapid Systematic Review and Meta-Analysis
61	Bin Abdulhak et al.	Angiotensin Converting Enzyme Inhibitors and Angiotensin Receptor Blockers and Outcome of COVID-19: A Systematic Review and Meta-analysis
62	Vakili et al.	Critical complications of COVID-19: A descriptive meta-analysis study
63	Yonas et al.	Effect of heart failure on the outcome of COVID-19 — A meta-analysis and systematic review

S3 Table. Country of origin of the primary studies reported in 56 systematic reviews

Primary study origin from the reporting 56 systematic reviews	(n=1575)
China	938 (59.6%)
USA	202 (12.8%)
Italy	110 (7.0%)
France	54 (3.4%)
Spain	50 (3.2%)
South Korea	40 (2.5%)
United Kingdom	36 (2.2%)
Multinational	30 (1.9%)
Netherland	12 (0.8%)
Turkey	12 (0.8%)
Iran	9 (0.6%)
Brazil	7 (0.4%)
Denmark	7 (0.4%)
Israel	7 (0.4%)
Switzerland	7 (0.4%)
Belgium	6 (0.4%)
Germany	6 (0.4%)
Japan	4 (0.2%)
Finland	3 (0.2%)
Hong Kong	3(0.2%)
Kuwait	3 (0.2%)
Australia	2 (0.1%)
French Territories	2 (0.1%)
Greece	2(0.1%)
India	2(0.1%)
Korea	2(0.1%)
Mexico	2(0.1%)
Thailand	2(0.1%)
Trinidad & Tobago	2(0.1%)
United Arab Emirates	2(0.1%)
Austria	1(0.1%)

Bolivia	1(0.1%)
Cameroon	1(0.1%)
Canada	1(0.1%)
Egypt	1(0.1%)
Ireland	1(0.1%)
Luxemburg	1(0.1%)
Pakistan	1(0.1%)
Poland	1(0.1%)
Singapore	1(0.1%)
South Africa	1(0.1%)

S1 Fig. The month of publication of primary studies and the country of origin

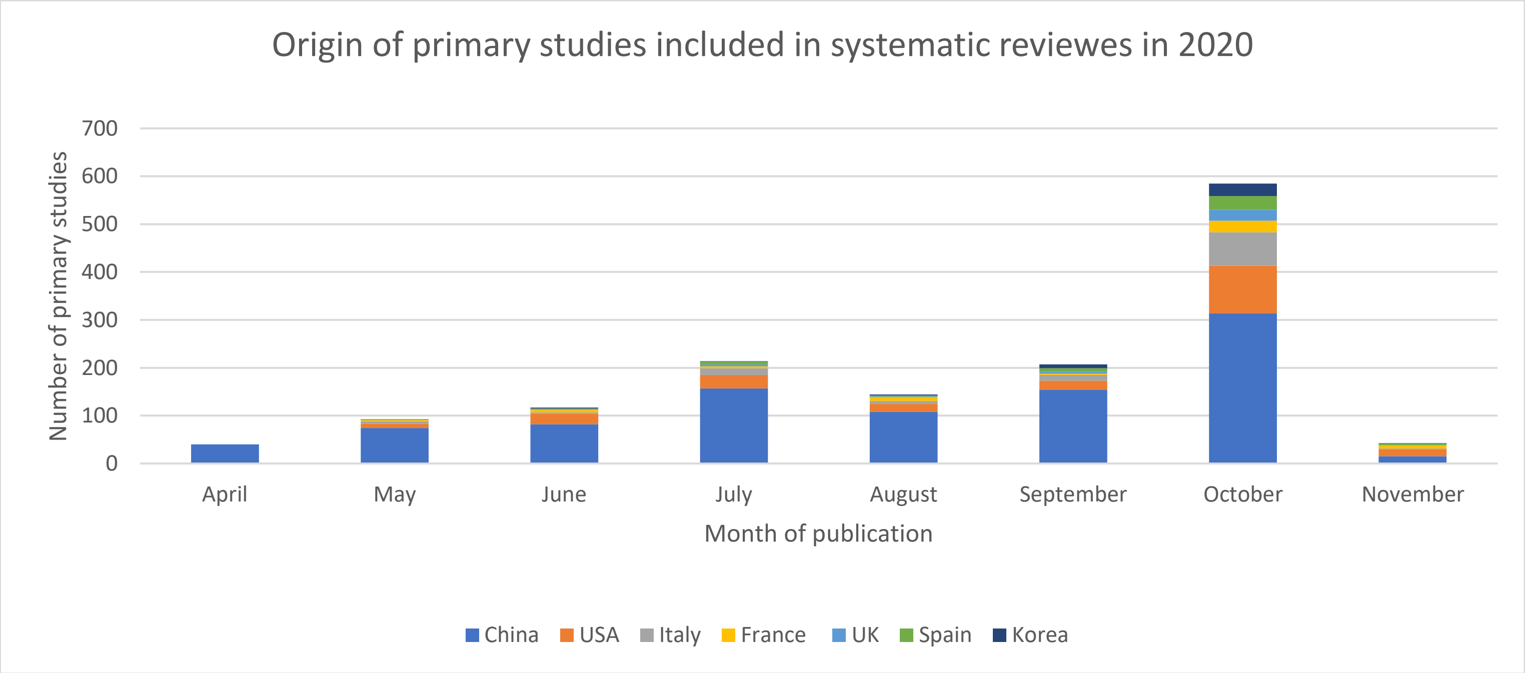

S2 Fig. Risk of Bias Assessment for 63 studies- ROBIS

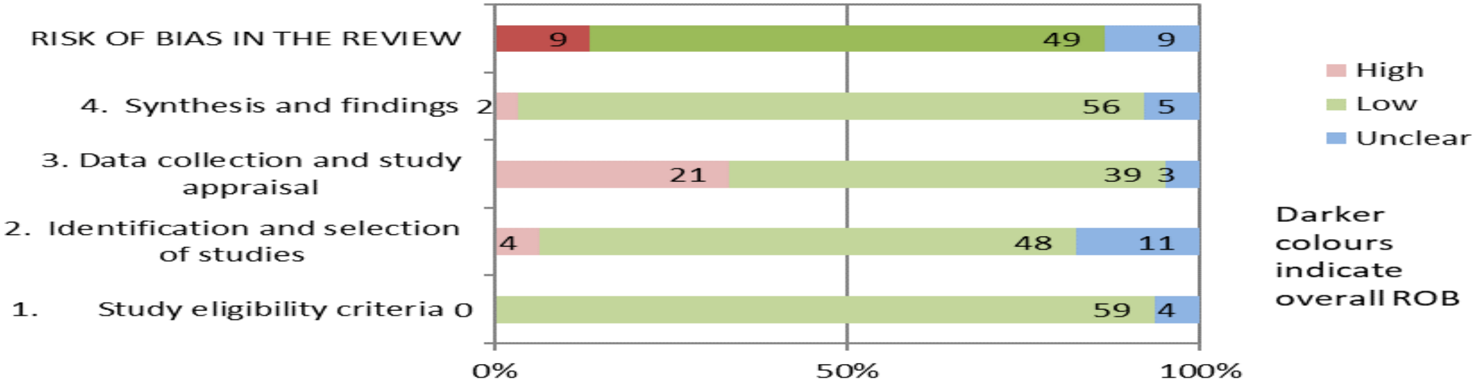

S3 Fig. Frequency of cardiac manifestations in COVID-19 patients

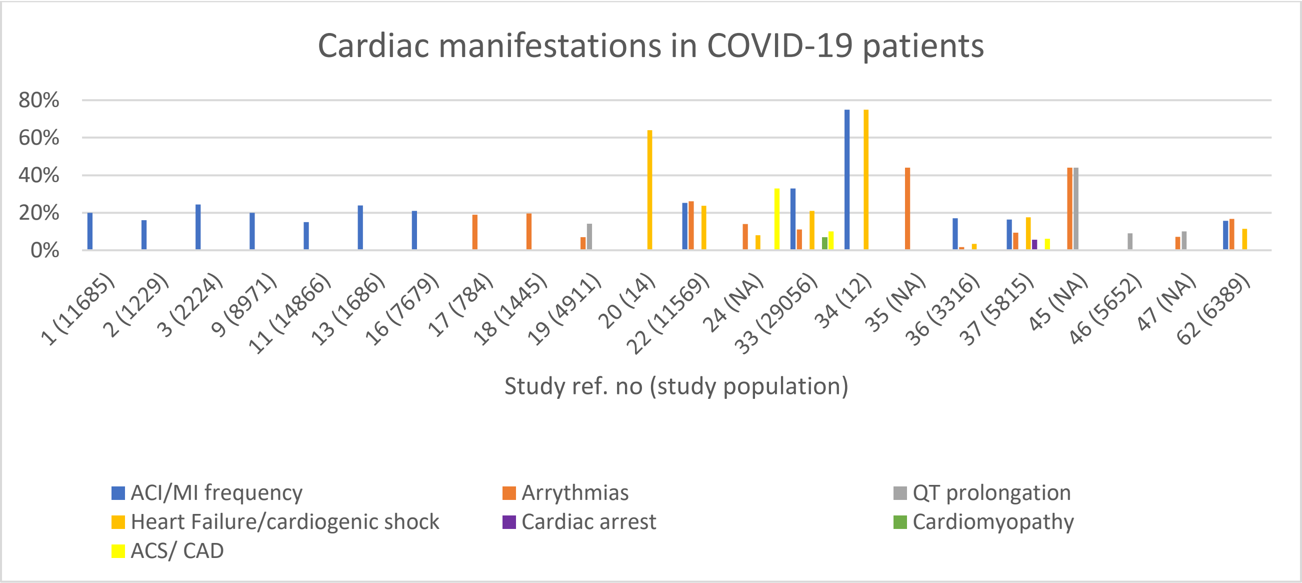

ACI/MI: Acute cardiac injury/Myocardial injury, ACS/CAD: Acute Coronary Syndrome/ Coronary Artery Disease

S4 Table. Studies addressing acute cardiac injury and myocardial injury in COVID-19 patients

Study Author.	Sample Size	Pre-existing cardiac disease in study population	ACI/MI frequency	ACI/MI severe vs non-severe/mild ds (OR)/(RR)	ACI/MI and mortality (OR)/ (RR)
Bavishi et al.	11685	NA/NR	20%	-	-
De Lorenzo et al.	1229	NA/NR	16%	-	-
Zou et al.	2224	NA/NR	24%	-	-
Huang et al.	5328	NA/NR	-	OR 13.5 [3.6, 50.5]	-
Li et al	4189	NA/NR	-	RR 6.0 [3.0, 11.8]	RR 3.8 [2.1, 7.0]
Luo et al.	129380	NA/NR	-	OR 6.6[3.7, 11.6]	OR 17.0 [7.9, 36.4]
Li et al.	3118	45-67%	15% - 44%	-	OR 21.2 [10.2, 43.9]
Prastilumkum et al.	8971	NA/NR	20%	-	-
Potere et al.	14866	9.4%	15%	-	-
Zeng et al.	5726	NA/NR	-	-	RR 4.9 [3.8, 6.2]
Zuin et al.	1686	NA/NR	23.90%	-	OR 22.5 [16.1, 31.4]
Santosa et al.	2389	NA/NR	-	RR 13.8 [5.5, 34.5]	RR 8.0 [5.1, 12.3]
Dalia et al.	5967	NA/NR	-	RR 8.5 [3.6, 20.0]	RR 8.5 [3.6, 20.0]
Gu et al.	7679	NA/NR	21%	RR 5.7 [3.7, 8.8]	
Momtazmanesh et al.	11569	NA/NR	25.30%	OR 6.3 [4.2, 9.8]	OR 19.6 [10.3, 37.5]
Shoar et al.	3257	NA/NR	-	-	OR 20.3 [7.8, 53.3]
Martins-Filho et al.	1141	NA/NR	-	-	RR 8.9 [4.2, 19.3]
Shashirian et al.	29056	NA/NR	33%	-	-
Singh et al.	12	NA/NR	75%	-	-
Sardinha et al.	3316	13.08%	17.09%	-	-
Kunutsor et al.	5815	14.6%	16.30%	-	-
Vakili et al.	6389	NA/NR	15.68%	-	-

S5 Table. Studies addressing other CV manifestations in COVID-19 patients: heart failure, cardiogenic shock, cardiac arrest, cardiomyopathy, acute coronary syndrome, coronary artery disease

Ref. No.	Heart Failure/cardiogenic shock-frequency	Cardiac arrest	Cardiomyopathy	ACS/ CAD
20	64%. (71% cardiogenic and 29% mixed cardiogenic and septic shock).	-	-	-
22	23.7% [19.3, 28.0]	-	-	-
24	8%	-	-	33%
28	HF as a risk for mortality RR = 5.1 [2.5, 10.7]	-	-	-
33	HF 21%, in ICU 20%. associated with mortality OR: 11.73 [5.17, 26.6]	-	7%	CHD 10%, CAD in ICU 20%.
34	75% (9/12 cases) HF	-	100%	-
36	3.43%	-	-	-
37	17.6%,	5.7%	-	ACS 6.2%
62	11.50% [3.45, 22.83]	-	-	-
46	-	0.3%	-	-
63	new-onset HF in hospitalized patients OR 11.67 [6.96, 19.56]	-	-	-

HF: Heart Failure, ACS/CAD: Acute Coronary Syndrome/Coronary Artery Disease, ICU: Intensive care unit

S6 Table. Studies reporting elevated cardio-biomarkers and the association with increased severity and mortality in COVID-19 patients

Ref. No.	Troponin I/Troponin	CK-MB	NT-BNP	LDH	D-dimer
1	17% patients	-	-	-	-
3	-	-	NT-BNP elevated in cardiac injury patients (SMD, 5.40)	-	-
5	Severe disease asso. with higher mean troponin (SMD 0.53)	Mean CK-MB higher in severe vs less severe cases (WMD 1.16)	Mean NT-proBNP higher in severe vs less severe cases (WMD 430.2)	-	-
6	elevated levels had significantly higher risks of severe disease, ICU admission, and death (RR 5.57, 95% CI 3.04 to 10.22, P < 0.001; RR 6.20, 95% CI 2.52 to 15.29, P < 0.001; RR 5.64, 95% CI 2.69 to 11.83, P < 0.001).	elevated CK-MB- non-severe disease/non-ICU and severe disease/ICU groups was 14.1% and 45.7%. Elevated levels at a higher risk of developing severe disease/ ICU admission (RR 3.24). Severe disease/ICU group at higher risk of developing an elevated CK-MB level vs nonsevere disease/non-ICU group (RR 3.24).	significantly higher in the elevated TnI group than in the non-elevated TnI group (SMD 1.63)	Elevated in 29.7% in the non-severe disease/ non-ICU group vs 60.1% severe disease/CU group. Elevated levels were significantly at increased risk of developing severe disease/ICU admission (RR 2.20)	-
8	pooled effect of these studies (unadjusted OR 21.15, 95% CI 10.19 to 43.94, p<0.001; heterogeneity: I2=70.5%, p=0.001) showed that patients with elevated troponin levels had a significant higher mortality risk than those with normal troponin levels.	-	-	-	-
10	high levels asso. With mortality (WMD=26.35 pg/mL, 95% CI=14.54–38.15, p<0.001),serum levels cardiac troponin I (weighted mean difference= 4.05 pg/mL, 95% CI = –0.20 to 8.30, p = 0.062, I2= 0.0%, pheterogeneity = 0.591) had no significant association with severity of the disease.	high serum levels (WMD=2.60 U/L, 95% CI=1.32–3.88, p<0.001) were associated with a significant increase in the severity of COVID-19. creatine kinase (weighted mean difference = 48.10 U/L, 95% CI = 0.27 to 95.94, p= 0.049, I2 = 85.0%, pheterogeneity = 0.001) were associated with a significant increase in the mortality of COVID-19.	-	High levels associated with a significant increase in the severity of COVID (WMD =108.86 U/L, 95% confidence interval (CI)=75.93–141.79, p<0.001). higher serum levels associated with increase mortality (WMD=213.44 U/L, 95% CI=129.97–296.92, p<0.001),	-

12	-	-	increased in cardiac injury patients, NT-pro BNP (SMD = 1.75) compared with non-CI	-	increased in cardiac injury patients (SMD = 0.89) compared with non-CI
14	higher in patients with primary + secondary outcome (mean difference 10.38 pg/mL)	-	-	-	-
15	higher in fulminant group but not statistically significant. MD 77.93 pg/mL (p=0.067).	significantly higher in severe group, levels of CKMB MD 1.98 ng/mL (p=0.012)]	significantly higher in severe group MD 1141.73 pg/mL (p=0.011)]	-	-
20	Elevated in 91% of myocarditis cases	elevated in 17% of myocarditis cases	Elevated in 50% of myocarditis cases	-	-
21	Elevated in 100% myocarditis cases	Elevated in 100% myocarditis cases	Elevated in 100% myocarditis cases	-	-
22	Significantly higher in deceased group (SMD = 2.96). Elevation observed in approximately one quarter of patients (25.3%)	elevated CK-MB 66.2% patients. higher in severe disease (SMD = 0.36).	increased ~40%. estimated pooled frequencies of elevation of NT-pro BNP were equal to 46.5%. NT-pro BNP (SMD = 1.13) significantly higher in the deceased group.	increased ~40%. (SMD = 1.54) significantly higher in the deceased group. pooled frequency equal to 41% patients. LDH levels higher in ICU or severe groups (SMD = 0.75)	increased ~40%. estimated pooled frequencies of elevation of D-dimer was 41.5%
23	-	-	higher in non-survivor group (SMD 0.75). Elevated NT-proBNP was associated with increased mortality (RR 3.63)	-	-
24	Elevated 17% (278/1659	CK-MB elevated 12% (133/1148).	elevated in 28% (106/380)	-	-
25	Mortality associated with elevation of cardiac troponin I (OR = 25.5, p = <0.0001)	-	serum level of BNP or pro-BNP was significantly elevated in the non-survivor group compared to the survivor group.	Mortality associated with elevation of LDH (OR = 11.8, p = 0.03)	-
26	on admission elevated levels (+44.2 ng/L, 95% CI, 19.0-69.4; P = .0006) btw fatal and survivors. significantly higher in nonsurvivors (+44.2 ng/L, P = .0006)	-	elevated in non-survivors (+903 pg/mL, P < .00001).	-	D-dimer (+4.6 µg/mL, 95% CI, 2.8-6.4; P < .00001) on admission between fatal and survivors
27	severe disease asso. with higher mean values of TnI (-0.54 [-0.72, -0.36]) (ng/mL),	mean difference of CK-MB in ng/mL between the severe and non-severe groups was found to be significant at -1.55, 95% CI [-2.23, -0.88] while it was not the case for the mean difference of CK-MB in units/L between the two groups (-4.75, 95% CI [-13.31, 3.82].	significant mean differences between the non-severe group and the severe group. NT-BNP (-815.7 [-1073.97, -557.42]) (pg/mL),	significant mean differences between the non-severe group and the severe group. LDH (-176.59 [-224.11, -129.06]) (units/L),	significant mean differences between the non-severe group and the severe group-dimer (-1.4 [-2.04, -0.77]) (mcg/mL),

28	large increase (SMD = 1.0, 95% CI 0.8 to 1.2) in non-survivors. Elevated hs-cTnI values above the 99th percentile URL associated with 8-fold increase in the risk of in-hospital death (RR = 8.0, 95% CI 2.2 to 28.5).	large increase in CK-MB (SMD = 1.0, 95% CI 0.2 to 1.8) in non-survivor patients.	large increase in NT-proBNP (SMD = 1.1, 95% CI 0.7 to 1.4) in non-survivor patients.	-	-

CK-MB: Creatine Kinase-MB, LDH: Lactate dehydrogenase, pro-BNP: pro Brain Natriuretic Peptide, OR: Odds ratio, RR: Relative risk, SMD: Standard mean deviation, WMD: Weighted mean deviation.

S7 Table. Hypertensive patients on RAAS inhibitors and COVID-19

Ref. No.	HTN, RAASI and testing positive	HTN, RAASI and hospitalization	HTN, RAASI and severe disease	HTN, RAASI and length of hospitalization	HTN, RAASI and mortality
48		OR 0.84 [0.58, 1.22]	OR 0.88 [0.68, 1.14]	MD -0.71	OR 0.77 [0.54, 1.12]
53			RR 0.73 [0.56-0.96]		RR 0.73 [0.56-0.96]
56			OR 0.76 [0.52, 1.12]		OR 0.64 [0.45, 0.89]
57	OR 0.87 [0.73, 1.04]		OR 0.64 [0.40, 1.02]		OR 0.93 [0.61, 1.42]
59					OR 0.63 [0.46, 0.86]
60	OR 1.00 [0.90, 1.12]		OR 0.73 [0.51, 1.03]		OR 0.57 [0.37, 0.87]

RAASI: Renin angiotensin aldosterone inhibitors, HTN: Hypertension, OR: Odds ratio

S8 Table. Studies addressing pathophysiology of cardiac involvement in COVID-19 patients

Study Ref. No.	Pathophysiology
1	immune-mediated inflammation plays a key role in the pathogenesis of COVID-19. innate and adaptive anti-viral immune response is vital in fighting the invading virus, on the other hand a robust and persistent anti-viral immune response may elicit an intense hyperinflammatory response akin to cytokine storm and cause damage to the host cells. The plausible mechanisms of myocardial injury include: 1) hyperinflammation and cytokine storm mediated through pathologic T cells and monocytes leading to myocarditis 2) respiratory failure and hypoxemia resulting in damage to cardiac myocytes, 3) down regulation of ACE2 expression and subsequent protective signaling pathways in cardiac myocytes, 4) hypercoagulability and development of coronary microvascular thrombosis, 5) diffuse endothelial injury and 'endotheliitis' in several organs, including the heart as a direct consequence of SARS-CoV-2 viral involvement and/or resulting from host inflammatory response and, 6) inflammation and/or stress causing coronary plaque rupture or supply-demand mismatch leading to myocardial ischemia/infarction (MI).
29	Confidently correlate a high hsTI with severe disease and death. It appears to be due to myocardial injury rather than thrombosis
30	more likely that cardiac injury in patients occurs by inflammation rather than direct infection
31	Myocyte injury occurs when metabolic demands induced by viral inflammation increase the challenge for a weak heart. Neither direct viral invasion of the virus to heart tissue nor lymphocytic infiltration consistent with myocarditis has been reported from the pathological analysis. Remarkably, myocyte necrosis was observed in autopsy analysis of patients from New Orleans, which suggests that SARS-CoV-2 could invade the pericytes and cause micro-circulation dysfunction. Cardiomyopathy is assumed to be a late complication of severe COVID-19; meanwhile, in few case reports, myocarditis was observed as a primary manifestation. The invasion of the viruses in the bloodstream mediated by ACE 2 receptors highly distributed in the heart and endovascular system stimulates CRS. Myocyte apoptosis could occur as a consequence of subsequent infiltration of neutrophils, and imbalanced T helper response. It is presumed that immune-mediated response is the main pathogenic mechanism in cardiomyopathy related to COVID-19. Increased sympathetic nervous system activity due to myocarditis and pro-inflammatory state are contributing factors to the development of cardiac rhythm abnormalities. Apart from these factors, hypoxia, hypotension, ACE 2-receptors downregulation, drug toxicity/interaction could also lead to developing or aggravating arrhythmic complications in patients with COVID-19.
32	Involvement of cardiomyocytes cannot be ruled out as there are reports of hypertrophied cardiomyocytes along with inflammatory infiltrates, focal edema, interstitial hyperplasia, fibrosis, degeneration, necrosis and signs of lymphocytic myocarditis. Besides ultrastructural changes like swelling of myocardial fibers, myocardium also shows presence of CD4 T cells along with other inflammatory infiltrates. However, pre-existing morbidity cannot be ruled out like hypertension-associated myocardial hypertrophy and past ischemic injury.<br><br>Endomyocardial biopsy from non-ischemic heart showed endocarditis and inflammation of interstitial tissue and viral particles have been observed in interstitial cells with damaged cell membrane, but myocytes were apparently normal with no viral particles. Viral particles may be absent while other signs of inflammation may be present within the cardiac tissue like leucocyte infiltration and presence of CD4+ T cells indicating indirect injury to the cells by the virus.
